# Utility of artificial intelligence in the diagnosis and management of keratoconus: a systematic review

**DOI:** 10.3389/fopht.2024.1380701

**Published:** 2024-05-17

**Authors:** Deniz Goodman, Angela Y. Zhu

**Affiliations:** Bascom Palmer Eye Institute, Miller School of Medicine, University of Miami, Miami, FL, United States

**Keywords:** keratoconus, corneal ectasia, artificial intelligence, machine learning, deep learning

## Abstract

**Introduction:**

The application of artificial intelligence (AI) systems in ophthalmology is rapidly expanding. Early detection and management of keratoconus is important for preventing disease progression and the need for corneal transplant. We review studies regarding the utility of AI in the diagnosis and management of keratoconus and other corneal ectasias.

**Methods:**

We conducted a systematic search for relevant original, English-language research studies in the PubMed, Web of Science, Embase, and Cochrane databases from inception to October 31, 2023, using a combination of the following keywords: artificial intelligence, deep learning, machine learning, keratoconus, and corneal ectasia. Case reports, literature reviews, conference proceedings, and editorials were excluded. We extracted the following data from each eligible study: type of AI, input used for training, output, ground truth or reference, dataset size, availability of algorithm/model, availability of dataset, and major study findings.

**Results:**

Ninety-three original research studies were included in this review, with the date of publication ranging from 1994 to 2023. The majority of studies were regarding the use of AI in detecting keratoconus or subclinical keratoconus (n=61). Among studies regarding keratoconus diagnosis, the most common inputs were corneal topography, Scheimpflug-based corneal tomography, and anterior segment-optical coherence tomography. This review also summarized 16 original research studies regarding AI-based assessment of severity and clinical features, 7 studies regarding the prediction of disease progression, and 6 studies regarding the characterization of treatment response. There were only three studies regarding the use of AI in identifying susceptibility genes involved in the etiology and pathogenesis of keratoconus.

**Discussion:**

Algorithms trained on Scheimpflug-based tomography seem promising tools for the early diagnosis of keratoconus that can be particularly applied in low-resource communities. Future studies could investigate the application of AI models trained on multimodal patient information for staging keratoconus severity and tracking disease progression.

## 1 Introduction

### 1.1 Keratoconus

Keratoconus is a progressive corneal ectasia characterized by stromal thinning and corneal steepening, ranging in severity from asymptomatic subclinical disease to severe corneal scarring requiring corneal transplantation for visual rehabilitation (1). As the condition progresses, patients may experience decreased visual acuity, photophobia, and image distortion (1). A meta-analysis study reported that younger patients and patients with a baseline maximum keratometry steeper than 55 D were more likely to experience disease progression (2). Patients with a medical history involving allergic eye disease or atopic conditions were also more likely to experience keratoconus progression (3). The pathophysiology of keratoconus involves reduced keratocyte density, loss of stromal lamellae with fibroblast degradation, redistribution of collagen, and increased proteolysis leading to a breakdown in structural integrity (4). This condition most commonly occurs between the second and third decade of life and has an estimated global prevalence of 1.38 per 1000 individuals (5, 6). The global prevalence of keratoconus varies across populations but has been previously estimated to range between 0.2-4790 per 100,000 individuals, with the lowest reported in Russia (5). The highest prevalence rates have been reported in Asian and Middle Eastern communities (5).

Multiple ancillary testing modalities have been used in conjunction with clinical examination for the diagnosis of keratoconus. Placido-based videokeratoscopy and ultrasonic central pachymetry were previously used in the diagnosis and severity staging of keratoconus (7). Modern Placido disc-based corneal topography devices are still popular, as they provide information about corneal curvature, surface irregularities, and aberrations by generating color-coded maps (1, 8). More recently, Scheimpflug-based corneal tomography imaging has allowed for greater analysis of the cornea by generating three-dimensional representations of the anterior segment to provide information about corneal thickness as well as the anterior and posterior cornea (7, 9). Corneal tomography is now the gold standard method for corneal ectasia diagnosis, and the Pentacam® (OCULUS, Arlington, WA, USA) is one of the most common Scheimpflug-based corneal tomography devices (9, 10). Another adjunct imaging technology used for evaluation of corneal ectasias is anterior-segment optical coherence tomography (AS-OCT), which generates cross-sectional corneal images that can be used to identify asymmetry in corneal thinning, posterior curvature, and epithelial/total corneal thickness via epithelial mapping (1). Corneal biomechanics, including corneal hysteresis and deformation amplitude, can also be used to diagnose early stages of keratoconus through an association with central corneal thickness (11).

Early keratoconus diagnosis is important for successful management and prevention of disease progression (12). Corneal cross-linking (CXL) is a procedure developed in 2003 that promotes bond formation between corneal collagen fibrils using riboflavin and ultraviolet-A light, which has demonstrated excellent long-term efficacy in reducing progression of keratoconus but has a threshold of procedural safety for the thinnest pachymetry value that can be treated (12). As CXL alone does not significantly improve visual outcomes, early detection and disease management can also allow for enhanced visual rehabilitation of patients without requiring keratoplasty, including options of hard contact lenses, intracorneal ring segment implantation, intraocular collamer lens implantation, and CXL combined with laser ablative procedures (e.g. photorefractive keratectomy) (12).

### 1.2 Models of artificial intelligence

The application of artificial intelligence (AI) in the diagnosis and management of ophthalmic diseases has been rapidly increasing since the 1970s (13). AI associates a particular outcome with variables of different weights, and trained models can be applied in establishing disease diagnosis, determining management, and predicting the prognosis of patients (14). Machine learning (ML) is a subset of AI that uses a training dataset for tasks but does not require programming (15). Features from the input data, which may include imaging and patient demographic information, are used to form a feature vector which then serves as the starting point for the ML model (16). Supervised ML is trained on a set of inputs with correct outcome labels available (17). In contrast, unsupervised ML models receive inputs without outcome labels (18). ML techniques include logistic regression, decision tree, random forest, support vector machine, and multilayer perceptron (**Table 1**) (15, 16).

**Table 1:** Description, use, and examples of machine learning techniques in medical literature.

ML Technique	Description	Preferred Use	Example of Clinical Application
Logistic Regression	Prediction based on predetermined input parameters (16)	Classifier (16)	Distinguish benign and malignant breast nodules using radiomic features from ultrasound (19)
Decision Tree	Uses binary decisions on selected features and criterion to form the final decision (16)	Multi-class problems, (20) defining groups based on a combination of features and similar outcomes (21)	Predicting classes of membrane proteins (integral, peripheral, and lipid-anchored) (22)
Random Forest	Uses randomization to form several decision trees and combines the output of the decision trees with voting or averaging (23)	Prediction tasks, interactions between predictor variables (23)	Prediction of tumor relapse, secondary malignant tumor, or all- cause death among patients with breast cancer after neoadjuvant chemotherapy (24)
Support Vector Machine	Training set is divided into 2 classes based on a hyperplane and a formula for the hyperplane is determined (16)	Binary classification problems (20)	Discriminating malignant versus normal gastric tissue (25)
Multilayer Perceptron	Feedforward neural network with the following layers: input, output, hidden. (26) Each layer contains communicating and connected nodes similar to synapsing neurons. (27) Nodes have an activation function that acts on the input to produce the output. (27) The weight of connections between nodes is based on ability to produce outcome (27)	Pattern classification, recognition, prediction, approximation, class associations (26, 27)	Predict mortality among patients with respiratory cancer in the intensive care unit (28)

Deep learning (DL) is a subset of machine learning that includes convolutional neural networks and does not require manual feature extraction from the research team (15, 29). DL models are composed of neural network layers that represent operations, and the output of one layer serves as the input of the next layer (30). Convolutional neural networks consist of convolution layers, pooling layers, and a fully connected network layer (16). Back propagation, normalization of input, dropout, and residual networks may be used to reduce error, reduce overfitting, and improve training (16).

The application of AI models is evaluated through several measures, which most commonly include accuracy, sensitivity, specificity, and area under the receiver operating curve (AUC or AUROC). The output of AI models in the majority of included studies is a class prediction (i.e. keratoconus versus healthy). Accuracy is the proportion of predictions that are true positives and negatives among all predictions. Sensitivity of a class is the proportion of true positives among all predictions of that class. Specificity describes the rate of true negatives (31). AUC ranges from 0 to 1 and is the area under the curve of the function modeled by sensitivity and 1-specificity. This provides a measure of diagnostic accuracy, with higher scores (closer to 1) representing greater accuracy (32).

### 1.3 Ophthalmic applications of artificial intelligence

The application of AI models in ophthalmology has been widely studied, particularly for posterior segment diseases including diabetic retinopathy, glaucoma, age-related macular degeneration, and retinopathy of prematurity (ROP) (33, 34). Ng et al. reported that AI algorithms for diabetic retinopathy may be closest to application in clinical medicine (33). DL systems are able to detect diabetic retinopathy, predict disease progression, and predict diabetic macular edema using fundus images (33). There also exist DL algorithms to detect glaucoma and predict progression using fundus photographs, optical coherence tomography, and Humphrey visual fields (33). AI systems are particularly useful for detecting ROP from fundus imaging given that there is grading variation among experts, and some low-resource countries have heavy disease burden with limited access to specialist care (33). DL algorithms have demonstrated high AUC, sensitivity, and specificity for identifying retinopathy of prematurity requiring further management (33).

There has also been an increase in reports evaluating the application of AI systems in detecting anterior segment diseases, including keratoconus, infectious keratitis, cataract, and pterygium using anterior segment photographs and AS-OCT images (35). AI has also been used to screen patients for post-corneal transplant rejection as well as to grade cataracts (35). However, several studies regarding the application of AI in anterior segment disease diagnosis and management were limited by a small sample size with low heterogeneity (35).

As shown, clinical utilization of AI models in ophthalmology has rapidly increased in the past 7 years since the first reports of DL systems trained for screening of retinal pathologies (36, 37). Due to the greater variety of anterior segment imaging and variable use in evaluating different conditions, widespread adoption of ML/DL algorithms for corneal pathology has not yet occurred. However, as imaging-based evaluation of keratoconus has now become standard of care due to improved technology, this study aims to provide a systematic review on the current state of utilizing AI and ML/DL platforms in the diagnosis, evaluation, management, and prognosis of keratoconus and corneal ectasias.

## 2 Methods of literature search

### 2.1 Search strategy

Using the Preferred Reporting Items for Systematic reviews and Meta-Analyses (PRISMA) framework as a guide, a systematic review technique was used to evaluate studies describing artificial intelligence and keratoconus. The search strategy was created based on the population, interventions, comparators, outcomes, and study design (PICOS) architecture, resulting in the study question: “Is artificial intelligence a sensitive and specific tool for the diagnosis and management of keratoconus or other corneal ectasias compared to clinical diagnosis and management led by ophthalmologists?” The systematic search was conducted using the PubMed, Web of Science, Embase, and Cochrane databases from inception to October 31, 2023, to select full-length, English articles in peer-reviewed journals. The MeSH keywords included in the search strategy were keratoconus, corneal ectasia, artificial intelligence, machine learning, and deep learning, with all combinations of these terms searched.

### 2.2 Inclusion and exclusion criteria

**Figure 1:**
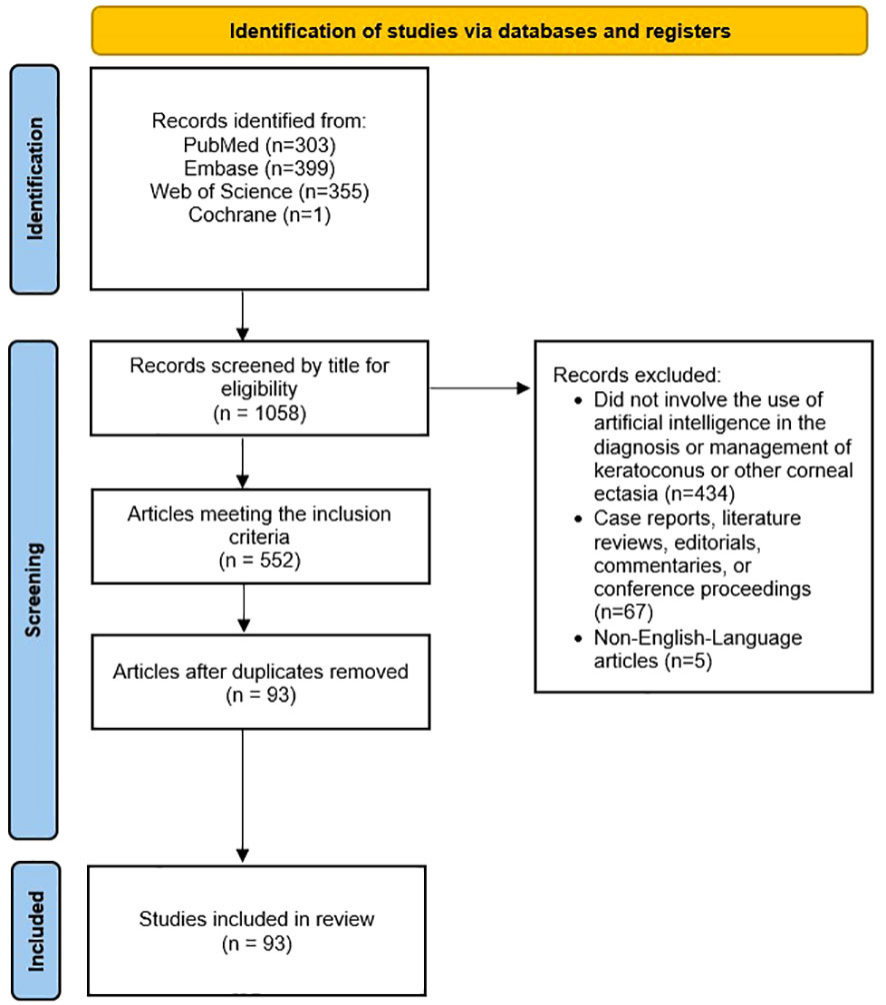
Preferred Reporting Items for Systematic Reviews and Meta-Analyses (PRISMA) diagram of the study selection methodology for this review.

Original, English-language research articles published in peer-reviewed journals regarding the use of any AI, ML or DL model in the evaluation of the diagnosis, pathophysiology, severity, clinical progression, or evaluation of the response to management of keratoconus and other corneal ectasias were included in this study. Case reports, editorials, commentaries, conference abstracts, and literature reviews were excluded. Non-English-language articles were also excluded. Publications were not restricted by year. **Figure 1** displays PRISMA diagram of the study selection methodology (38).

### 2.3 Data extraction

The following variables were collected for each included study:

- Type of artificial intelligence
- Input used for training
- Output
- Ground truth or reference standard
- Dataset size (number of images, eyes, and patients, when applicable and available)
- Availability of algorithm/model
- Source and availability of dataset
- Major study findings

### 2.4 Quality assessment

The Quality Assessment of Diagnostic Accuracy Studies-2 (QUADAS-2) tool was used to assess the risk of bias and applicability concerns of studies regarding the diagnosis, severity, and clinical grading of keratoconus and other corneal ectasias (39). The Quality Assessment of Prognostic Accuracy Studies (QUAPAS) tool was used to assess the risk of bias and applicability concerns of studies regarding the prediction of disease progression and response to treatment/management (40). A quality assessment for studies regarding the etiology and pathogenesis was not completed due to the absence of a validated assessment tool for this type of study.

## 3 Results

The search strategy yielded 93 original research studies. These studies are summarized in **Table 2**, and we discuss some key studies in the following Results section. There were no concerns regarding the applicability of any studies given the broad nature of our review question and strict application of the inclusion and exclusion criteria.

**Table 2:** Overview of the number of studies, types of AI models implemented for each study aim, and quality assessment of studies included in this review. The types of AI models include multilayer perceptron (MLP), convolutional neural network (CNN), generative adversarial network (GAN), traditional machine learning (ML), ensemble, and natural language processing (NLP).

Study Aim	Number of Studies (n=93)	Types of AI Models Implemented	Risk of Bias Assessment Summary
Diagnosis	61	MLP, CNN, GAN, ML, ensemble	Patient Selection: 75.4% High, 6.6% Low, 18.0% Unclear; Index Test: 62.3% Low, 37.7% Unclear; Reference Standard: 96.7% Low, 3.3% Unclear; Flow and Timing: 82.0% Low, 18.0% Unclear
Etiology & Pathogenesis	3	NLP, ML	Not applicable
Severity & Clinical Grading*	16	MLP, ML, ensemble, CNN,	Patient Selection: 69.2% High, 7.7% Low, 23.1% Unclear; Index Test: 92.3% Low, 7.7% Unclear; Reference Standard: 100% Low; Flow and Timing: 76.9% Low, 23.1% Unclear
Keratoconus Progression	7	MLP, CNN	Participants: 14.3% High, 57.1% Low, 28.6% Unclear; Index Test: 100% Low; Outcome: 100% Low; Flow and Timing: 85.7% Low, 14.3% Unclear; Analysis: 100% Low
Response to Treatment/Management**	6	MLP, ML, CNN	Participants: 20.0% High, 40.0% Low, 40.0% Unclear; Index Test: 100% Low; Outcome: 100% Low; Flow and Timing: 100% Low; Analysis: 80% Low, 20% Unclear

*3/16 studies were not included in the risk of bias assessment because the QUADAS-2 tool was not applicable to their study design.

**1/6 studies were not included in the risk of bias assessment because the QUAPAS tool was not applicable to the study design.

### 3.1 Diagnosis of keratoconus

Among the included studies, AI was most frequently used for the diagnosis of keratoconus and other ectasias, particularly in subclinical cases (n = 61, **Supplementary Table 1**) (31, 41–100). The earliest study using artificial intelligence to detect keratoconus was published in 1994 by Maeda et al. (65) This study used eight indices from TMS-1 videokeratoscope data (Computed Anatomy Inc., New York City, NY, USA) to detect keratoconus among a set of eyes with normal corneas and corneas with various diagnoses (65). The linear discriminant function generated a Keratoconus Prediction Index value that was used to classify eyes as “keratoconus” or “nonkeratoconus” based on a cutoff value (65, 100). The model demonstrated sensitivities of 100% and 89% with three false-positives and one false-positive in the training and validation set, respectively (65). More recently, Silverman et al. evaluated the use of stepwise linear discriminant analysis and neural networks in detecting keratoconus using corneal epithelial and stromal thickness maps from Artemis 1® (StarFish Medical, Victoria, BC, Canada) very high frequency ultrasound arc-scans (96). Eyes with keratoconus were reported to have focal epithelial and stromal thinning with a surrounding ring of epithelial thickening (96, 101).

The majority of the included studies regarding keratoconus or corneal ectasia diagnosis with AI used data from corneal topography (47, 51–53, 56, 57, 67, 73, 74, 77, 78, 83, 91, 92, 94, 100), Scheimpflug-based tomography (43, 45, 49, 54, 55, 59, 60, 62, 64, 66, 70, 76, 79, 80, 84, 86–88, 90, 95, 99), or optical coherence tomography (OCT) (31, 46, 50, 53, 68, 69, 72, 80, 84) as the input. For example, de Almeida et al. utilized 52 parameters from Pentacam® (OCULUS, Arlington, WA, USA) tomography to generate the Corneal Tomography Multivariate Index (CTMVI) using paraconsistent feature engineering and a support vector machine classifier (90). CTMVI was then used to discriminate between very asymmetric ectasia with normal topography and healthy corneas. When using these 52 features, CTMVI demonstrated a sensitivity, specificity, and AUC of 0.844, 0.874, and 0.926, respectively (90). After combining CTMVI and the Pentacam Random Forest Index, the model demonstrated a sensitivity and specificity of 0.99 and 0.84, respectively. Other input for AI-based keratoconus or corneal ectasia diagnosis included data from corneal deformation videos (42, 55, 71, 97), air-puff tonometry (50), corneal endothelial images from specular microscopy (81), and lateral segment photographs (93).

Beyond diagnosing subclinical keratoconus or keratoconus, Ahn et al. demonstrated that AI can be used to screen patients in a primary care setting that may need further evaluation with corneal topography for keratoconus. Using input parameters of subjective visual impairment (based on a patient survey), visual acuity, intraocular pressure, and autokeratometry parameters, they compared the performance of five previously-reported AI models (102–106). The ensemble model with soft voting method demonstrated superior performance with this task with a sensitivity of 90.5% and 96.4% in the internal and external test datasets, respectively (44). Intraocular pressure and mean corneal power were the most highly ranked in the feature importance analysis (44). A soft voting ensemble classifier has been previously used to predict major adverse cardiovascular events among patients with acute coronary system (107).

In the absence of a large dataset of eyes with keratoconus, Lavric et al. and Subramanian et al. used SyntEyes KTC to generate a sufficiently large set of corneal topography images for model training (57, 97). The SyntEyes KTC model was developed by Rozema et al. using Scheimpflug tomography, ocular biometry, and wavefront data of 145 eyes with keratoconus (108). Abdelmotaal et al. used a conditional generative adversarial network (CGAN) called pix2pix to produce images of keratoconus eyes (95). The pix2pix CGAN is composed of a Generator that uses the input image to form the output image, and a Discriminator that determines the similarity of the generated image to an image from the original dataset or an image from the Generator (95). The authors reported that the model produced subjectively and objectively plausible images of keratoconus, early keratoconus, and normal eyes (95). They trained a VGG-16 deep convolutional neural network on combinations of original images and images synthesized by the pix2pix CGAN. The VGG-16 model trained with all original images and synthesized images demonstrated the highest accuracy of 99.56% in discriminating early keratoconus, keratoconus, and normal eyes (95).

### 3.2 Etiology & pathogenesis of keratoconus

Only three studies have investigated the utility of AI in exploring the genetic etiology and mechanical pathogenesis of keratoconus (**Supplementary Table 2**) (109–111). Hosoda et al. conducted a genome-wide association study (GWAS) of central corneal thickness using IBM’s Watson for Drug Discovery AI technology. They found the STON2 rs2371597 and SMAD3 rs12913547 loci to be involved in keratoconus development. STON2 and SMAD3 have roles in extracellular matrix (ECM) remodeling, so these variants may contribute to the stromal ECM changes described in keratoconus (109, 112). The authors noted that additional GWAS may be used to identify pathways driving keratoconus development (109). Wang et al. identified 8 differentially expressed genes (AREG, BBC3, DUSP2, MAP3K8, SMAD7, CDKN1A, JUN, and LIF) between patients with and without keratoconus using the random forest model, support vector machine model, and generalized linear model (110). These genes may affect cell mitosis (AREG), macrophage dysfunction (BBC3), cell cycle arrest (CDKN1A), apoptosis (DUSP2, CDKN1A), and proliferation, differentiation, and death (JUN) (110). The authors concluded that abnormal cell proliferation, differentiation, and autophagy pathways may be involved in keratoconus development (110).

Given the reported association between mechanical eye rubbing and pathogenesis of keratoconus, Nokas et al. built a wrist-mounted sensor that used an accelerometer, gyroscope, and machine learning algorithms to detect eye rubbing activity and remind the user to cease such activity (111). With limited AI-based studies on this topic, future research could further investigate susceptibility genes and utility of behavior modification in keratoconus pathogenesis.

### 3.3 Severity & clinical grading of keratoconus

Sixteen original research articles identified in this review assessed severity or other clinical features of patients with keratoconus (**Supplementary Table 3**) (104, 113–127). Among studies evaluating keratoconus severity, the ground truth included staging based on topography findings (104, 113, 118, 120, 125, 127), tomography findings (116), the Ectasia Screening Index (114), Keratoconus Severity Index (119), and Amsler-Krumeich criteria (120, 121, 126). Chen et al. compared six convolutional neural network models trained with one or a combination of four color-coded corneal tomography maps (axial, anterior elevation, posterior elevation, and pachymetry) as well as a majority voting strategy model to predict the presence and stage of keratoconus (121). The model trained with all four maps demonstrated the best AUC in distinguishing healthy from keratoconus eyes (121). Interestingly, the majority voting model and the model using the back elevation map demonstrated the highest AUC for discriminating healthy and stage 1 keratoconus eyes, as well as stage 1 and stage 2 keratoconus eyes, respectively (121).

Dong et al. and Dos Santos et al. both used deep learning models to segment corneal OCT scans (122, 123). Dong et al. used a corneal segmentation algorithm to measure the thickness of epithelial and stromal tissue with an error of less than 4 microns (123). The OCT images were taken by the Optovue RTVUE 100 device (Optovue, Inc., Fremont, CA, USA). They reported that as keratoconus progressed, total corneal thickness decreased, particularly temporal and inferior to the pupil center (123). Epithelial thickness also decreased as keratoconus severity increased (123). However, keratoconic eyes with stromal scarring demonstrated a larger epithelial thickness with irregular variations (123). Dos Santos et al. developed CorneaNet, a neural network to segment OCT images of eyes with and without keratoconus and produce thickness maps of the epithelium, Bowman layer, and stroma (122). The image scans were acquired on a custom-built, ultra-high resolution OCT system (128). CorneaNet achieved an accuracy of 99.56% with this task, but the authors noted that accuracy may be limited by network architecture, image noise, as well as insufficient and incorrect training data (122).

### 3.4 Keratoconus progression

Seven studies investigated AI-based prediction of disease progression in eyes with keratoconus (**Supplementary Table 4**) (129–135). The majority of these studies only provided binary categorization of keratoconus eyes as progressive or nonprogressive. Kundu et al. developed two AI models, based on either tomographic changes or clinical risk factors, to identify risk factors underlying keratoconus progression and label patients with either likely disease “progression” or “no progression.” (129) They found that elevated serum immunoglobin E (IgE), systemic allergies, eye rubbing, and serum vitamin D level were important characteristics in the evaluation for the risk of keratoconus progression (129). Patients categorized into the progression group had a significantly higher serum IgE compared to those categorized into the no progression group (129). Eye rubbing may also be due to ocular irritation, fatigue, and stress (129). Eye rubbing reduces keratocyte density and modifies intraocular pressure, which can contribute to keratoconus development (129). The results of this study were limited in their generalizability given that the study was based only on an Asian Indian group of patients (129).

Alternatively, Kamiya et al. used a deep learning model to predict keratoconus progression with color-coded maps from AS-OCT (133). They reported that after adjusting for age, the accuracy of their algorithm improved from 0.794 to 0.849 (133). However, this study was limited by the absence of external validation and confirmation of repeatability, small sample size, possibly inaccurate keratoconus diagnosis, and the effect of contact lenses (133).

### 3.5 Response to treatment/management

Six studies featured the use of AI in characterizing the response to different treatment modalities, such as by predicting postoperative outcomes or the need for future intervention (**Supplementary Table 5**) (136–141). Both Valdés-Mas et al. and Lyra et al. employed machine learning to predict postoperative refractive outcomes after intracorneal ring implantation, including corneal curvature, astigmatism, asphericity, and keratometry (138, 140). Liu et al. utilized machine learning to predict postoperative visual acuity and keratometry two years after corneal crosslinking (139). While these studies demonstrate the potential of AI models to aid in disease prognostication after different treatments, validation studies are necessary prior to their widespread generalization and adoption.

## 4 Discussion

Keratoconus is a corneal ectatic disease that can be diagnosed through several imaging modalities, including corneal topography and tomography (1). Furthermore, early detection of keratoconus and other corneal ectasias with prompt management can help slow disease progression and reduce the risk for vision loss or need for a corneal transplant (12). Therefore, AI systems that can process images and generate predictions may be used to reduce the rate of missed or delayed diagnoses, thereby improving patient outcomes. We conducted a systematic review to evaluate the state of AI systems that have been applied in the diagnosis and management of keratoconus and other corneal ectasias among adult patients.

We found that the majority of original research studies involving the clinical application of AI algorithms among patients with keratoconus are based on disease diagnosis. The most current and highest impact studies in the field include Al-Timemy et al., Tan et al., Ambrosio et al., Kuo et al., Kamiya et al., and Chen et al. (42, 52, 72, 82, 88, 121) The majority of these studies used a convolutional neural network AI model, which is a DL method that does not require manual feature extraction from the research team unlike ML algorithms (15, 29). Most of these studies also used Scheimpflug-based corneal tomography scans or indices as their model’s input. This may be explained by the ability of Scheimpflug-based corneal tomography to provide additional information about the posterior cornea, which has been shown to exhibit ectatic changes earlier than the anterior cornea in keratoconus, allowing for earlier diagnosis (142). Additionally, the Belin ABCD classification system (A: anterior radius of curvature, B: back (posterior) radius of curvature, C: minimal corneal thickness, and D: best spectacle distance visual acuity) was developed to monitor disease progression evident on the posterior corneal surface in the absence of anterior corneal surface changes (142). Given that the aforementioned studies demonstrated relatively high diagnostic accuracy of DL algorithms trained on tomography input, further refinement and validation of AI systems evaluating information from Scheimpflug-based tomography may be adopted in clinical practice to streamline the identification of patients with subclinical keratoconus that may benefit from close monitoring for progression to clinical disease. Additionally, a mobile-based application with this technology can be used to develop a screening program for subclinical keratoconus and other corneal ectasia in low-resource countries.

AI models have been less commonly used in other contexts, such as identifying genetic susceptibility and predicting disease progression. Unlike disease diagnosis, these contexts may rely on model input that involves more subjective elements, such as demographic, environmental risk factors, and other clinical factors. For example, Kundu et al. recently developed an AI model using the random forest algorithm to predict progressive keratoconus using clinical and ocular surface risk factors determined from a patient questionnaire (129). Their system demonstrated an AUC of 0.81, and 76% of cases classified as progressive by an AI model trained on tomographic changes were also classified as progressive by the clinical risk factors AI model (129). Within each nondiagnostic context, the AI systems demonstrated relative variability in their performance in completing classification or prediction tasks, and further research is needed to draw conclusions regarding the application of AI models in nondiagnostic contexts for keratoconus. In the future, validated AI models trained on environmental and clinical risk factors could be particularly useful in predicting disease progression to identify patients at risk for severe disease. These identified patients may potentially benefit from prophylactic corneal crosslinking to strengthen corneal integrity and reduce risk of progression.

Some studies included in this review were limited by their design. For example, only one specialist determined the presence of keratoconus for the reference standard of some diagnostic accuracy studies, including Almeida Jr. et al., Lucena et al., Lopes et al., Chandapura et al., Cohen et al., Zéboulon et al., Mosa et al., and Zaki et al. (45, 47, 49, 53, 54, 75, 87, 93) This could have resulted in biased classification of eyes. Additionally, some studies included a relatively low sample size of study groups, which reduced the power of the study. Ahn et al. included only 69, 39, and 43 patients in the subclinical keratoconus study group in the training, internal, and external datasets, respectively (44). Other studies, such as Cohen et al., do not include parameters for demographic information, such as age, sex, or race/ethnicity which can affect the baseline corneal curvature and thickness (54, 143–145).

The strengths of this study lie in the comprehensiveness of clinical contexts included in this systematic review. While previously published reviews regarding the application of AI in keratoconus and other corneal ectasia focus on diagnostic accuracy (146–149), we also included studies regarding grading disease severity, predicting disease progression, understanding etiology and pathogenesis, and predicting response to treatment or management. However, our review has several limitations. To begin with, except for nine studies published prior to 2013, the majority of studies included in our review were published within the last decade. This may be explained by the fact that the application of ML/DL in medicine, including ophthalmology, has grown in popularity more recently (150). Furthermore, we searched a limited number of medical databases, including PubMed, Embase, Web of Science, and Cochrane. Studies meeting our eligibility criteria but not indexed in these databases may have been excluded from this review. While we completed a qualitative data extraction among included studies, we were unable to conduct a meta-analysis with statistical methodology given the contextual and methodological variation between studies and their AI systems. Lastly, some studies included in this review were based on specific patient populations with low diversity, which decreases the generalizability of this review’s findings.

With the incorporation of ML/DL AI algorithms and advancements in corneal imaging technology, the potential ability of clinicians to detect and treat keratoconus at earlier stages to prevent disease progression is promising. AI can also be used to determine ectasia severity, identify susceptibility genes, categorize the keratoconus as progressive or non-progressive, and predict response to surgical management. These applications are particularly important for low-resource nations which may have a scarcity of cornea specialists. In these underserved areas, the incorporation of validated AI models could help reduce the rate of missed or delayed corneal ectasia diagnoses. For example, validated smartphone applications could allow for longitudinal at-home screening of keratoconus among patients with risk factors for the condition. Looking ahead, some clinical applications of AI include using multimodal patient data (a combination of corneal images, demographic information, and environmental risk factors) as the input to determine the keratoconus stage and track disease progression.

Additionally, AI can be used in the surgical planning of some therapeutic options for keratoconus that are growing in popularity, such as ray-tracing-guided transepithelial photorefractive keratectomy with accelerated crosslinking, which may help reduce refractive overcorrection and stromal tissue ablation (151). Mazzotta et al. used a tissue-preservation algorithm involving ray tracing among 38 patients with stable keratoconus undergoing this procedure (151). They reported that the algorithm and surgical treatment significantly improved visual outcomes in their cohort (151). This demonstrates that AI-based algorithms could personalize surgical planning to improve postoperative outcomes, so the implementation of these algorithms for other surgical procedures for visual rehabilitation in keratoconus patients should be investigated.

## Data availability statement

The original contributions presented in the study are included in the article/**Supplementary Material**. Further inquiries can be directed to the corresponding author.

## Author contributions

DG: Investigation, Writing – original draft, Writing – review & editing. AYZ: Conceptualization, Methodology, Supervision, Writing – original draft, Writing – review & editing.
