# Supplementary material for: Utility of artificial intelligence in the diagnosis and management of keratoconus: a systematic review
Source: Front Ophthalmol (Lausanne). 2024 May 17;4:1380701. doi: 10.3389/fopht.2024.1380701 (PMC11182163; doi:10.3389/fopht.2024.1380701)
Supplement: Supplementary file 1 [file Table_1.docx]

**Supplemental Table 1.** Original research studies for the application of artificial intelligence in the diagnosis of keratoconus or other corneal ectasia.

| **Author, Year** | **Type of AI** | **Input used for training** | **Output** | **Ground Truth/Reference Standard** | **Dataset size** | **Availability of Algorithm/Model** | **Availability of Dataset** | **Major Study Results** | **Risk of Bias Assessment** |
| --- | --- | --- | --- | --- | --- | --- | --- | --- | --- |
| Mohammadpour et al., 2021(41) | Four classifiers (Pentacam Belin/Ambrósio enhanced ectasia total deviation value, Topographic Keratoconus Classification, Sirius Phoenix, and OPD-Scan III Corneal Navigator | Belin/Ambrósio enhanced ectasia total deviation value used pachymetry progression and elevation maps. Sirius Phoenix used corneal thickness, curvature, elevation, and aberrometry indices. OPD-Scan III Corneal Navigator used 19 corneal indices. Topographic Keratoconus Classification was based on the Amsler-Muckenhirn criteria. | Categorized eyes as keratoconus, subclinical keratoconus, or normal. | Expert categorization as normal, keratoconus, or subclinical keratoconus based on retinoscopy, slit lamp biomicroscopy, visual acuity, topography, and keratometry. | 212 eyes of 212 patients:  92 normal eyes  52 subclinical keratoconus eyes  68 keratoconus eyes | N/A | Single Center (Noor Eye Hospital, Iran). Data availability not specified. | - Sirius Phoenix demonstrated the best performance with a sensitivity and specificity of 84.62% and 90.0%, respectively, in detecting subclinical keratoconus. - Sirius Phoenix also had the highest accuracy in discriminating keratoconus from subclinical keratoconus. | Patient Selection: High  Index Test:  Low  Reference Standard: Low  Flow and Timing: Low |
| Tan et al., 2022(42) | Five-layer feedforward neural network | Biomechanical parameters including time of the first applanation, deformation amplitude at the highest concavity, central corneal thickness, and radius at the highest concavity from corneal deformation videos | Detected keratoconus versus normal eyes. | Clinical diagnosis of keratoconus based on slit lamp biomicroscopy, topography, and visual acuity. | 177 keratoconus eyes of 143 patients  177 normal eyes of 118 patients  Of the 354 corneal deformation videos, 276 were used for training and testing while 78 were used for external validation. | N/A | Single Center (Refractive Surgery Center at the Tianjin Eye Hospital, China). Data availability not specified. | - The five-layer feedforward neural network model demonstrated an accuracy, sensitivity, specificity, and precision of 98.7%, 97.4%, 100%, and 100%, respectively, in diagnosing keratoconus using the external validation set. - This model’s performance was found to be superior to that of support vector machine and computer-aided diagnosis. | Patient Selection:  Unclear  Index Test:  Unclear  Reference Standard:  Low  Flow and Timing:  Low |
| Xu et al., 2022(43) | Four machine-learning algorithms (eXtreme Gradient Boosting, Light Gradient Boosting Machine, Logistic Regression, and Random Forest) and a convolutional neural network model (KerNet) | Objective indices from Pentacam HR | Distinguished clinically unaffected eyes among patients with asymmetric keratoconus from keratoconus and normal eyes. | Keratoconus was diagnosed based on the Collaborative Longitudinal Evaluation of Keratoconus Study criteria. Asymmetric keratoconus was diagnosed based on slit lamp biomicroscopy, retinoscopy, ophthalmoscopy, topography, visual acuity, and history of contact lens use, eye surgery, and trauma. | 1108 eyes of 1108 patients:  430 normal eyes  231 asymmetric keratoconus eyes  447 keratoconus eyes  664 eyes in the training set, 222 eyes in the test set, and 222 eyes in the external validation set | N/A | Single Center (Zhejiang University School of Medicine, China). Data availability not specified. | - Achieved an accuracy and AUC of 94.12% and 0.983, respectively, in diagnosing asymmetric keratoconus in the validation set. | Patient Selection:  Low  Index Test:  Unclear  Reference Standard:  Low  Flow and Timing: Low |
| Ahn et al., 2022(44) | Three fully connected neural network models and two ensemble models with hard and soft voting methods | Subjective visual impairment, visual acuity, intraocular pressure, autokeratometry, mean corneal power, and differences in these values between the right and left eyes from dentified who were recommended corneal topography | Recommended corneal topography | Clinical diagnosis based on Pentacam topography, slit lamp biomicroscopy, and visual acuity. | Training dataset (1518 patients): 999 normal, 69 subclinical keratoconus, 450 clinical keratoconus  Internal test dataset (457 patients): 383 normal, 38 subclinical keratoconus, 36 clinical keratoconus  External test dataset (638 patients): 527 normal, 43 subclinical keratoconus, 68 clinical keratoconus | N/A | Multicenter (Severance Hospital, Yonsei University College of Medicine and the Eyejun Ophthalmic Clinic, Korea). Dataset available upon request. | - The ensemble model with soft voting method demonstrated superior performance with a sensitivity of 96.4% in the external dataset. | Patient Selection: Low  Index Test:  Unclear  Reference Standard:  Low  Flow and Timing: Low |
| Almeida Jr et al., 2022(45) | Multiple logistic regression analysis | Age and 21 corneal parameters | Produced the Boosted Ectasia Susceptibility Index (BESTi) to differentiate patients in the following groups: (1) very asymmetric ectasia and with normal corneal topography and tomography (VAE-NTT), (2) corneas with clinical keratoconus, and (3) healthy corneas | Cornea specialist categorized patients into keratoconus and very asymmetric ectasia with normal corneal tomography. | 2893 eyes of 2893 patients:  187 VAE-NTT eyes  2296 healthy eyes  410 ectasia eyes  Data divided into internal validation (50%) and external validation (50%) sets. | N/A | Multicenter ((1) Visum Eye Center, Brazil and (2) Rio Claro Eye Institute, Brazil). Data availability not specified. | - BESTi outperformed the Belin-Ambrósio Deviation Index and Pentacam random forest index in distinguishing patients with VAE-NTT from patients with healthy corneas with an AUC, sensitivity, and specificity of 0.91, 86.02%, and 83.97%, respectively. | Patient Selection:  High  Index Test:  Unclear  Reference Standard:  Low  Flow and Timing:  Low |
| Kundu et al., 2023(46) | Random forest AI model | Curvature, wavefront aberrations, thickness distributions, and volume of epithelium and total cornea from Placido and optical coherence tomography | Distinguished healthy eyes from eyes with keratoconus or very asymmetric ectasia. | Clinical diagnosis based on medical record review, slit lamp examination, and tomography. | 527 healthy eyes of 527 patients  144 very asymmetric ectasia eyes of 144 patients  454 keratoconus eyes of 454 patients | N/A | Single Center (Narayana Nethralaya, India). Data not available. | - Demonstrated an AUC, accuracy, recall, and precision of 0.997, 99.1%, 98.7%, and 99.1%, respectively, for identifying eyes with keratoconus. - Achieved an AUC, accuracy, recall, and precision of 0.976, 95.5%, 71.5%, and 91.2%, respectively for identifying eyes with very asymmetric ectasia. | Patient Selection:  Low  Index Test:  Unclear  Reference Standard:  Low  Flow and Timing:  Low |
| Lucena et al., 2021(47) | TopEye iOS application using a convolutional neural network | 240 images of each topographical pattern | Classified corneal topography as spherical, regular and symmetrical astigmatism, regular and asymmetrical astigmatism, or irregular astigmatism (keratoconus) | Specialist manually outlined the corneal contour and labeled the pattern displayed in the image | 1172 corneal topography images:  275 spherical  302 regular and symmetrical astigmatism  295 regular and asymmetrical astigmatism  300 irregular astigmatism (keratoconus) images  Data divided into training (81.91%) and testing (18.09%) sets. | N/A | Data availability not specified. | - TopEye correctly classified 94.81% of images and demonstrated a sensitivity and specificity of 95.00% and 98.68%, respectively, in detecting keratoconus. | Patient Selection: High  Index Test:  Unclear  Reference Standard:  Low  Flow and Timing:  Low |
| Issarti et al., 2019(48) | Computer aided diagnosis system that combines a feedforward neural network with a Grossberg-Runge Kutta architecture | Anterior and posterior elevation as well as corneal thickness data | Detected clinical and suspect keratoconus from normal eyes | Clinical diagnosis based on slit lamp findings, tomography maps, and ocular history. | 851 eyes of 851 patients:  312 eyes with bilateral normal tomography  90 keratoconus suspect eyes  220 mild keratoconus eyes  229 moderate keratoconus | N/A | Single Center (Antwerp University Hospital, Belgium). Data availability not specified. | - Achieved a sensitivity, specificity, and accuracy of 97.78%, 95.56%, and 96.56%, respectively, and demonstrated superior performance in detecting suspect keratoconus compared to the Belin/Ambrósio Deviation and Topographical Keratoconus Classification. | Patient Selection:  High  Index Test:  Unclear  Reference Standard:  Low  Flow and Timing:  Low |
| Lopes et al., 2018(49) | Machine learning models with regularized discriminant analysis, support vector machine, naïve Bayes, neural networks, and random forest | Tomography data from Pentacam | Distinguished eyes with corneal ectasia from healthy eyes | Corneal specialist categorized cases (rotating Scheimpflug corneal and anterior segment tomography) into keratoconus and very asymmetric ectasia groups | Training set: 2980 patients with stable laser-assisted in situ keratomileusis (LASIK), 182 keratoconus eyes of 182 patients, 71 eyes of 45 patients who developed post-LASIK ectasia  Test set: 298 patients with stable LASIK, 376 eyes of 188 patients without operations with very asymmetric ectasia with normal topography in one eye and clinically diagnosed ectasia in the other eye | N/A | Multicenter ((1) Optical Express, United Kingdom, (2) Instituto de Olhas Renato Ambrósio, Brazil, (3) Vincieye Clinic, (4) Price Vision Group, USA, (5) London Vision Clinic, United Kingdom). Data availability not specified. | - The Pentacam Random Forest Index achieved high sensitivities of detecting very asymmetric ectasia with normal topography (85.2%) and post-LASIK ectasia (80%). | Patient Selection:  High  Index Test:  Low  Reference Standard:  Low  Flow and Timing:  Unclear |
| Lu et al., 2022(50) | AI models with random forest algorithm or neural networks | Spectral-domain optical coherence tomography (SD-OCT) and air-puff tonometry | Detected keratoconus versus normal eyes | Clinical diagnosis of keratoconus, forme fruste keratoconus, and early keratoconus based on slit lamp findings, Scheimpflug topography, and visual acuity. | 223 normal eyes of 223 patients  72 early keratoconus eyes of 72 patients  69 forme fruste keratoconus eyes of 69 patients  258 advanced keratoconus eyes of 258 patients  Data divided into training (70%) and validation (30%). | N/A | Single Center (Eye Hospital of Wenzhou Medical University, China). Data availability not specified. | - The AI model with the random forest algorithm achieved the best accuracy for detecting forme fruste keratoconus with an AUC, sensitivity, and specificity of 99%, 75%, and 94.74%, respectively. | Patient Selection:  High  Index Test:  Unclear  Reference Standard:  Low  Flow and Timing:  Unclear |
| Lavric et al., 2019(51) | KeratoDetect algorithm with a convolutional neural network | Corneal topography maps generated by SyntEyes KTC | Detected keratoconus versus normal eyes | Not reported | Training set: 1350 topographies  Validation set: 150 eyes  Test set: 200 healthy eyes, 200 keratoconus eyes | N/A | Generated by SyntEyes KTC. Data available in the manuscript. | - Achieved an accuracy of 99.33% in detecting keratoconus. | Patient Selection:  Unclear  Index Test:  Unclear  Reference Standard:  Low  Flow and Timing:  Low |
| Kuo et al., 2020(52) | 3 convolutional neural network models (VGG16, Inception V3, ResNet152) | AI models were pretrained. Input was color-coded topography maps. | Detected keratoconus versus normal eyes | Clinically diagnosed keratoconus based on slit lamp findings and corneal topography | 354 images of 354 eyes:  Training set: 134 keratoconus eyes, 120 normal eyes  Subclinical test: 28 subclinical keratoconus eyes  Test set: 36 keratoconus eyes, 30 normal eyes | N/A | Single Center (National Taiwan University Hospital, Taiwan). Data availability not specified. | - ResNet152 demonstrated the highest accuracy (0.958), sensitivity (0.944), specificity (0.972), and area under the receiver operating characteristics (AUROC) curve (0.995) in detecting keratoconus compared to the other AI models. | Patient Selection:  High  Index Test:  Low  Reference Standard:  Low  Flow and Timing:  Low |
| Chandapura et al., 2019(53) | 4 random forest models | Optical coherence tomography and Pentacam indices including keratometry, maximum curvature, and surface aberrations. | Detected forme fruste keratoconus, keratoconus, and normal eyes | Clinical diagnosis made by a refractive surgeon based on corneal topography of the anterior surface. | 221 normal corneas  72 forme fruste keratoconus corneas  116 keratoconus corneas | N/A | Single Center (Federal University of São Paulo, Brazil). Data availability not specified. | - The random forest model using the air-epithelium interface and epithelium-Bowman’s capsule interface was significantly better in detecting forme fruste keratoconus compared to the random forest model using Scheimpflug scans only. | Patient Selection:  High  Index Test:  Low  Reference Standard:  Low  Flow and Timing:  Low |
| Cohen et al., 2022(54) | Machine learning with random forest algorithms | Scheimpflug/Placido corneal tomography maps: axial curvature, elevation with best fit, elevation with fit toric asphere, and pachymetry | Classified eyes as keratoconus, suspect irregular, or normal. | Corneal specialist labeled images as normal, suspect irregular, or keratoconus based on Scheimpflug/Placido maps | 7104 images of 3787 eyes  Training set: 3508 normal images, 1099 suspect irregular images, 2194 keratoconus images  Test set: 580 normal images, 200 suspect irregular images, 420 keratoconus images | N/A | Single Center (Tel Aviv Sourasky Medical Center, Israel). Data availability not specified. | - The model combining keratoconus prediction indices of the Scheimpflug/Placido tomographer with 94 output parameters from the tomographer had an AUC, accuracy, sensitivity, and specificity of 0.969, 91.5%, 94.7%, and 89.8%, respectively, in distinguishing normal, suspect irregular, and keratoconus images. | Patient Selection:  High  Index Test:  Unclear  Reference Standard:  Low  Flow and Timing:  Low |
| Ambrósio Jr. et al., 2017(55) | Logistic regression analysis with forward stepwise inclusion, support vector machine, random forest | Corneal deformation response from Corvis ST and corneal tomography from Pentacam | Detected normal corneas versus corneas with corneal ectasia (keratoconus, very asymmetric ectasia with no surgery, very asymmetric ectasia with normal topography) | Clinical diagnosis based on slit lamp findings, and topography. | 480 normal eyes of 480 patients  204 keratoconus eyes of 204 patients  72 very asymmetric ectasia; 72 fellow eyes were categorized as very asymmetric ectasia with normal topography from 94 patients | N/A | Multicenter ((1) Instituto de Olhos Renato Ambrósio, Brazil and (2) Vincieye Clinic, Italy). Data availability not specified. | - The Tomographic and Biomechanical Index produced by the random forest AI model demonstrated a 100% sensitivity and specificity in diagnosing clinical ectasia with a cut-off value of 0.79. - This model had a greater AUROC compared to the use of Belin/Ambrósio Deviation or Corvis Biomechanical Index. | Patient Selection:  High  Index Test:  Unclear  Reference Standard:  Low  Flow and Timing:  Low |
| Gao et al., 2022(56) | KeratoScreen: Artificial neural networks using Zernike coefficients determined from corneal parameters | Zernike coefficient data sets from Pentacam topography maps (anterior corneal curvature, posterior corneal curvature, anterior corneal elevation, posterior corneal elevation, corneal thickness) | Classified eyes as subclinical keratoconus, keratoconus, or normal. | Clinical diagnosis based on slit lamp biomicroscopy and corneal topography. | 1040 corneal topography images of 208 eyes of 208 patients  70 normal eyes  48 subclinical keratoconus eyes  90 keratoconus eyes  Data divided into training (70%) and testing (30%) sets. | N/A | Single Center (Eye Hospital of Wenzhou Medical University, China). Data availability not specified. | - The use of Zernike coefficients determined from corneal thickness achieved the best sensitivity and precision rate for detecting subclinical keratoconus (93.9% and 96.1%, respectively) and keratoconus (97.6% and 95.1%, respectively). | Patient Selection:  High  Index Test:  Low  Reference Standard:  Low  Flow and Timing:  Unclear |
| Subramanian et al., 2022(57) | Pretrained convolutional neural network model for transfer learning and classifying images | Corneal topography images generated by the SyntEyes KTC model with and without segmentation with particle swarm optimization, discrete particle swarm optimization, or fractional order particle swarm optimization. | Classified eyes as keratoconus, subclinical keratoconus, or normal. | SyntEyes KTC model produced 500 images of each type. | Training set: 300 images each of normal, subclinical keratoconus, and keratoconus eyes  Test set: 200 images each of normal, subclinical keratoconus, and keratoconus eyes | N/A | Generated by SyntEyes KTC. Data available in the manuscript. | - Achieved an accuracy of 95.9% in classifying images segmented with particle swarm optimization. | Patient Selection:  Unclear  Index Test:  Unclear  Reference Standard:  Low  Flow and Timing:  Low |
| Lu et al., 2023(58) | Random forest or neural networks | Feature selection was conducted on Pentacam HR, RTVue-XR, and Corvis ST parameters. AI models were trained on features from Scheimpflug tomography, spectral-domain optical coherence tomography, and air-puff tonometry individually and combined. | Detected forme fruste keratoconus versus normal eyes | Clinical diagnosis made by 3 physicians based on slit lamp findings, Scheimpflug tomography, and visual acuity. | 271 normal eyes  84 forme fruste keratoconus eyes  85 early keratoconus eyes  159 advanced keratoconus eyes  Data divided into training (60%) and validation (40%) sets. | N/A | Single center (Eye Hospital of Wenzhou Medical University, China). Data availability not specified. | - The random forest model using features from spectral domain-optical coherence tomography and air-puff tonometry achieved the highest AUC for detecting forme fruste keratoconus (0.902) among combinations of two devices. | Patient Selection:  High  Index Test:  Unclear  Reference Standard:  Low  Flow and Timing:  Unclear |
| Cao et al., 2020(59) | 8 machine learningmethods: random forest, decision tree, logistic regression, support vector machine, linear discriminant analysis, multilayer perceptron neural network, lasso regression, k-nearest neighbor | Each machine learning method was trained on 11 parameters: age, gender, axial length, spherical equivalent, mean front corneal curvature, mean back corneal curvature, central corneal thickness, corneal thickness at the apex, corneal thickness at the thinnest point, anterior chamber depth, and corneal volume. | Detected subclinical keratoconus versus normal eyes | Clinical diagnosis based on corneal topography, slit lamp biomicroscopy, and retinoscopy. | 88 eyes of 88 patients:  49 subclinical keratoconus eyes  39 control eyes | N/A | Single Center (Royal Victorian Eye and Ear Hospital, Australia). Data availability not specified. | - The random forest method using five parameters (gender, spherical equivalent, front corneal curvature, corneal volume, and corneal thickness at the thinnest point) demonstrated the best AUC (0.97) for identifying subclinical keratoconus compared to other methods. | Patient Selection:  High  Index Test:  Low  Reference Standard:  Low  Flow and Timing:  Low |
| Smadja et al., 2013(60) | Machine learning algorithm: classification and regression tree | Parameters of eyes with a known classification. Tomography parameters were based on curvature, elevation, corneal wavefront, pachymetry, and biometry. | Classified eyes as normal, forme fruste keratoconus, or keratoconus | Classification based on corneal topography, tomography, and slit lamp findings. | 177 normal eyes of 95 patients  47 forme fruste keratoconus eyes of 47 patients  148 keratoconus eyes of 102 patients | N/A | Single center (University Hospital of Bordeaux, France). Data availability not specified. | - The automated decision tree classifier demonstrated high sensitivity and specificity for distinguishing keratoconus and normal eyes (100% and 99.5%, respectively), as well as for distinguishing forme fruste keratoconus and normal eyes (93.6% and 97.2%, respectively). | Patient Selection:  High  Index Test:  Low  Reference Standard:  Low  Flow and Timing:  Low |
| Karimi et al., 2018(61) | Artificial neural network with multi-step-ahead prediction algorithm | Corvis ST data and von Mises stress | Detected keratoconus versus normal eyes | Clinical diagnosis based on keratometry, retinoscopy, and slit lamp findings. | Training set: 40 healthy eyes, 40 keratoconus eyes  Verification set: 155 eyes of 155 patients | N/A | Single center (Basir Eye Hospital, Iran). Data availability not specified. | - Achieved greater than 95.5% accuracy. | Patient Selection:  High  Index Test:  Unclear  Reference Standard:  Low  Flow and Timing:  Low |
| Ruiz Hidalgo et al., 2017(62) | Keratoconus Assistant (machine learning) | 25 anterior and posterior surface topography and tomography parameters from Pentacam | Classified eyes as keratoconus, forme fruste or suspect keratoconus, postrefractive surgery, or normal/regular astigmatism based on a probability | Clinical diagnosis by corneal specialists from 2 centers (Fondation Rothschild and Antwerp University Hospital (UZA)). Rothschild specialists used slit lamp examination, and Orbscan topography. UZA specialists used Pentacam tomography. | 131 eyes of 102 patients separately classified by the two centers:  UZA classification: 54 keratoconus, 44 normal, 10 postrefractive surgery, 23 keratoconus suspect  Rothschild classification: 51 keratoconus, 61 normal, 11 postrefractive surgery, 8 keratoconus suspect | N/A | Single Center (Rothschild Foundation, France). Data availability not specified. | - Demonstrated a moderate agreement with Rothschild and UZA specialists in classifying cases (kappa = 0.594 and 0.563, respectively). | Patient Selection:  High  Index Test:  Low  Reference Standard:  Low  Flow and Timing:  Low |
| Maeda et al., 1995(63) | Model based on discriminant analysis and classification tree | 8 topographic indexes from corneal topographic maps from the TMS-1 videokeratoscope | Categorized topographic maps as keratoconus versus nonkeratoconus | Categorization based on previous diagnosis | 176 eyes of 125 patients:  44 topographic maps of keratoconus eyes  132 topographic maps of nonkeratoconus conditions (30 normal, 26 with-the-rule astigmatism, 20 contact lens-induced corneal warpage, 28 excimer laser photorefractive keratectomy, 19 penetrating keratoplasy, 9 pellucid marginal degeneration) | N/A | Single Center (Louisiana State University Eye Center, USA). Data availability not specified. | - Demonstrated a sensitivity and specificity of 98% and 99%, respectively, in distinguishing keratoconus versus nonkeratoconus maps. | Patient Selection:  High  Index Test:  Low  Reference Standard:  Low  Flow and Timing:  Unclear |
| Cao et al., 2021(64) | Random forest machine learning model | 15 Pentacam parameters selected from 1692 total parameters using principal component analysis | Detected subclinical keratoconus versus normal eyes | Clinical diagnosis made by keratoconus specialists and optometrists using slit lamp biomicroscopy, retinoscopy, and corneal tomography. | 145 subclinical keratoconus eyes of 141 patients  122 control eyes of 85 patients  Data divided into training (70%) and test (30%) sets. | Code for dimensionality reduction, model establishment, tuning, and evaluation is available on GitHub. | Single Center (Royal Victorian Eye and Ear Hospital, Australia). Data availability not specified. | - Demonstrated an accuracy and sensitivity, of 98% and 97%, respectively. | Patient Selection:  High  Index Test:  Low  Reference Standard:  Low  Flow and Timing:  Low |
| Maeda et al., 1994(65) | Combination of classification tree and linear discriminant function | 8 indices from TMS-1 videokeratocoscope data | Generated a cutoff value relative to the Keratoconus Prediction Index that was used to detect keratoconus pattern versus nonkeratoconus pattern. | 3 researchers classified maps using clinical records and topography | Training set: 100 eyes including 22 keratoconus eyes and 30 normal eyes  Validation set: 100 eyes including 28 keratoconus eyes and 30 normal eyes | N/A | Single Center (Louisiana State University Eye Center, USA). Data availability not specified. | - Demonstrated a sensitivity, specificity, and accuracy of 89%, 99%, and 96%, respectively, in the validation set. | Patient Selection:  High  Index Test:  Low  Reference Standard:  Low  Flow and Timing:  Low |
| Feng et al., 2021(66) | KerNet: convolutional neural network with multi-level fusion architecture | 2D Pentacam image slices of front surface curvature, back surface curvature, front surface elevation, back surface elevation, and pachymetry with ground truth labels | Classified eyes as keratoconus, subclinical keratoconus, or normal | Ground truth labels with correct classification | 335 normal eyes of 335 patients  172 subclinical keratoconus eyes of 172 patients  347 keratoconus eyes of 347 patients  Data divided into training (80%) and test (20%) sets. | Code available on GitHub. | Single center (Eye Center of the 2^nd^ Affiliated Hospital, Zhejiang University, China). Data availability not specified. | - Demonstrated a mean accuracy of 95.91% for detecting subclinical keratoconus and 98.25% for detecting keratoconus. | Patient Selection:  High  Index Test:  Unclear  Reference Standard:  Low  Flow and Timing:  Low |
| Al-Timemy et al., 2021(67) | 7 deep learning models based on EfficientNet-b0 (pretrained on ImageNet) for feature extraction, and support vector machine classifier for the classification task | Corneal topographic maps of anterior and posterior eccentricity, anterior and posterior elevation, anterior and posterior sagittal curvature, and corneal thickness maps | Classified eyes as suspected keratoconus, keratoconus, or normal. | 3 corneal specialists classified each eye based on corneal topography, keratometric curvature, oblique cylinder, central corneal thickness, front elevation map, back elevation map, and Belin/Ambrósio Enhanced Ectasia Display indices. | Development set (3794 images of 542 eyes from 280 patients): 204 normal eyes, 123 suspected keratoconus eyes, 215 keratoconus eyes  Validation set (1050 images of 150 eyes from 85 patients): 50 normal eyes, 50 suspected keratoconus eyes, 50 keratoconus eyes | N/A | Multicenter (Federal University of São Paulo–UNIFESP/EPM, Brazil, and Hospital de Olhos–CRO, Brazil). Dataset publicly available for research purposes. | - In the independent test dataset, the accuracy and AUC for distinguishing normal and keratoconus eyes was 92% and 0.99, respectively. - The accuracy and AUC for distinguishing normal, keratoconus, and subclinical keratoconus eyes was 68.7% and 0.81, respectively. | Patient Selection:  Unclear  Index Test:  Low  Reference Standard:  Low  Flow and Timing:  Low |
| Elsawy et al., 2021(68) | Deep learning neural network including a pretrained VGG19 | Anterior segment optical coherence tomography images with diagnosis labels | Detected dry eye syndrome, Fuchs’ endothelial dystrophy, and keratoconus | Clinical diagnosis made by one of 6 corneal specialists | 99360 images of 483 eyes of 286 patients:  19440 healthy images of 98 eyes of 58 patients  13320 Fuchs’ endothelial dystrophy images of 59 eyes of 35 patients  46800 keratoconus images of 240 eyes of 148 patients  19800 dry eye syndrome images of 86 eyes of 45 patients | N/A | Single center (Bascom Palmer Eye Institute, USA). Data available upon request. | - Achieved an AUROC of 0.99 for detecting keratoconus at the eye level, and 0.97 at the image level. | Patient Selection:  High  Index Test:  Low  Reference Standard:  Low  Flow and Timing:  Low |
| Fassbind et al., 2023(69) | Convolutional neural network based on the ConvNeXt architecture | Anterior axial refraction, posterior axial refraction, anterior elevation, posterior elevation, and pachymetry from anterior segment-optical coherence tomography scans labeled with the diagnosis | Detected healthy versus diseased corneas | Cornea experts labeled images with one of the following: healthy, keratoconus, post laser, keratoconus, post laser, keratoglobus, pellucid marginal corneal degeneration, other, and not appreciable | 1940 scans of 899 patients:  Training set: 1552 total scans including 719 healthy scans and 290 keratoconus scans  Validation set: 388 total scans including 185 healthy scans and 61 keratoconus scans  Test set: 242 total scans including 113 healthy scans and 112 keratoconus scans | N/A | Single center (Saarland University Hospital Clinic for Ophthalmology, Germany). Data available upon request | - Demonstrated a sensitivity and specificity of 98.46% and 91.96%, respectively, in detecting diseased corneas from normal corneas. - Among the test scans, the model demonstrated an accuracy of 92.56% in identifying keratoconus. | Patient Selection:  Unclear  Index Test:  Low  Reference Standard:  Low  Flow and Timing:  Low |
| Kovács et al., 2016(70) | Machine learning classifiers | Trained using the scaled conjugate gradient algorithm and bilateral Scheimpflug measurements | Detected healthy eyes versus fellow eyes of patients with unilateral keratoconus | Clinical diagnosis based on slit lamp examination and corneal topography. | 60 eyes of 30 patients with bilateral keratoconus  60 normal eyes of 30 patients  15 normal fellow eyes of 15 patients with unilateral keratoconus  Data divided into training (70%) and test (30%) sets. | N/A | Single Center (Semmelweis University, Hungary) | - The multilayer perceptron classifier trained with bilateral index of height decentration achieved the best accuracy (AUROC of 0.96) in distinguishing healthy eyes from normal fellow eyes of patients with unilateral keratoconus. - This classifier also demonstrated high accuracy in detecting keratoconus eyes with an AUROC of 0.99 using bilateral index of height decentration. | Patient Selection:  High  Index Test:  Low  Reference Standard:  Low  Flow and Timing:  Low |
| Leão et al., 2019(71) | Attribute selection and discriminant function | Classifiers were developed using one or more of the following: deflection amplitude, Corvis ST output parameters, thickness horizontal profile, and intraocular pressure | Detected healthy versus keratoconus eyes | Corvis Biomechanical Index | Development set: 195 healthy eyes, 136 keratoconus eyes  Validation set: 156 healthy eyes, 87 keratoconus eyes | N/A | Multicenter ((1) Instituto de Olhos Renato Ambrósio, Brazil and (2) Vincieye Clinic, Italy) | - Deformation amplitude compensated by intraocular pressure using the linear discriminant function demonstrated the best performance in detecting keratoconus with an AUC, sensitivity, and specificity of 0.954, 88.2%, and 97.4%, respectively. | Patient Selection:  Unclear  Index Test:  Unclear  Reference Standard:  Low  Flow and Timing:  Unclear |
| Kamiya et al., 2019(72) | 6 deep learning neural network using pretrained ResNet-18 | Each neural network was trained with one of the following color-coded anterior segment optical coherence tomography maps: anterior or posterior elevation, anterior or posterior curvature, total refractive power, and pachymetry maps | Output values indicated the presence and grade of keratoconus (0= normal, 1=grade 1, 2=grade2, 3=grade 3, 4=grade4) | Clinical diagnosis made by corneal specialists based on corneal tomography and slit lamp findings. Classification of keratoconus grade based on the Amsler-Krumeich system. | 304 keratoconus eyes (108 grade 1 eyes, 75 grade 2 eyes, 42 grade 3 eyes, 79 grade 4 eyes)  239 healthy eyes | N/A | Single Center (Miyata Eye Hospital, Japan). Data available upon request. | - The model trained on posterior curvature maps demonstrated the best accuracy in detecting keratoconus (0.993) and grading keratoconus stage (0.869). | Patient Selection:  High  Index Test:  Low  Reference Standard:  Low  Flow and Timing:  Low |
| Maeda et al., 1995(73) | Neural network | 11 indices from topographic maps and diagnostic labels for each map | Classified topographic maps | Human experts classified the topographic maps as one of the following: normal, with-the-rule astigmatism, mild/moderate/advanced keratoconus, postphotorefractive keratectomy, or postkeratoplasty | Training set: 108 maps including 12 mild keratoconus, 14 moderate keratoconus, and 13 advanced keratoconus maps  Test set: 75 maps including 8 mild keratoconus, 12 moderate keratoconus, and 9 advanced keratoconus maps | N/A | Single center (Louisiana State University Eye Center, USA) | - Achieved an accuracy of 80% in classifying maps in the test set. - The sensitivity for classifying mild, moderate, and advanced keratoconus was 63%, 92%, and 44%, respectively. | Patient Selection:  High  Index Test:  Low  Reference Standard:  Low  Flow and Timing:  Low |
| Prakash et al., 2023(74) | Machine learning | Topography metrics, derived metrics, and clinical diagnoses were used to train the model. | Detected keratoconus versus normal eyes | Clinical diagnosis made by two corneal specialists based on clinical signs and symptoms, slit lamp biomicroscopy, and Topographic Keratoconus Classification. | 290 keratoconus eyes  847 normal eyes  20% of data was used for an isolated test set, and the remaining data was divided into training (80%) and validation (20%) sets. | N/A | Single center (University of Pittsburgh School of Medicine, USA) | - The machine learning model using corneal asymmetry ratios from all 4 meridians demonstrated a sensitivity and specificity of 99% and 94%, respectively, in detecting keratoconus. | Patient Selection:  High  Index Test:  Low  Reference Standard:  Low  Flow and Timing:  Low |
| Zéboulon et al., 2020(75) | Convolutional neural network | Orbscan examinations of each class (normal, keratoconus, or history of refractive surgery) | Classified eyes as normal, keratoconus, or history of refractive surgery | Corneal tomography expert selected Orbscan examinations for each category. Keratoconus examination classification was based on patterns in the anterior curvature map. | 900 Orbscan examinations for each category (normal, keratoconus, history of refractive surgery) in the training set  100 Orbscan examinations for each category (normal, keratoconus, history of refractive surgery) in the test set | N/A | Single center (Rothschild Foundation, France). Data not available. | - Achieved a sensitivity and specificity of 100% and 100%, respectively, for detecting keratoconus, as well as an overall classification accuracy of 99.3%. |  |
| Ruiz Hidalgo et al., 2016(76) | Support vector machine algorithm | 22 Pentacam parameters selected using correlation-based hierarchical clustering | Classified eyes as keratoconus versus normal, forme fruste keratoconus versus normal, and within one of 5 groups (keratoconus, forme fruste keratoconus, astigmatic, postrefractive surgery, normal) | Keratoconus specialist and optometrist categorized eyes using patient history and topography maps. | 454 keratoconus eyes of 329 patients  67 forme fruste keratoconus eyes of 67 patients  28 astigmatic eyes of 18 patients  117 postrefractive surgery eyes of 62 patients  194 normal eyes of 194 patients | N/A | Single Center (Antwerp University Hospital, Belgium). Data availability not specified. | - Demonstrated high accuracy, sensitivity, and specificity for detecting keratoconus (98.9%, 99.1%, and 98.5%, respectively) or forme fruste keratoconus (93.1%, 79.1%, and 97.9%, respectively) from normal eyes. - When classifying eyes across all five groups, the model demonstrated an accuracy of 88.8%. | Patient Selection:  High  Index Test:  Low  Reference Standard:  Low  Flow and Timing:  Low |
| Souza et al., 2010(77) | Machine learning classifiers: support vector machine, multilayer perceptron, radial basis function | 11 parameters from Orbscan II maps | Detected keratoconus versus other eyes | Clinical diagnosis based on medical record and Orbscan II review | 172 normal maps  89 astigmatism maps  46 keratoconus maps  11 photorefractive keratectomy maps | N/A | Single Center (private practice of an author). Data availability not specified. | - The support vector machine, multilayer perceptron, and radial basis function classifiers achieved an AUROC of 0.99, 0.99, and 0.98, respectively, in distinguishing keratoconus from other patterns on Orbscan II maps. | Patient Selection:  High  Index Test:  Unclear  Reference Standard:  Low  Flow and Timing:  Unclear |
| Accardo et al., 2002(78) | Neural network | Indices from corneal topographic maps of both eyes or individual eyes of patients | Classified eyes as normal, keratoconus, or nonkeratoconus | Clinical diagnosis from previously classified maps, family history, and data from follow-up examinations | 120 normal maps of 60 patients  110 keratoconus maps of 55 patients  166 nonkeratoconus conditions maps of 83 patients | N/A | Single Center (Ophthalmological Unit of the Children’s Hospital ‘Burlo Garofolo’ of Trieste, Italy). Data availability not specified. | - Achieved a sensitivity and specificity of 100% and 98.6%, respectively, in detecting keratoconus. | Patient Selection:  High  Index Test:  Low  Reference Standard:  Low  Flow and Timing:  Low |
| Castro-Luna et al., 2021(79) | Decision tree and random forest machine learning techniques | Pentacam and Corvis parameters | Detected subclinical keratoconus versus normal eyes | Clinical diagnosis based on topography, corneal curvature, corneal thickness, and slit lamp biomicroscopy. | 61 healthy eyes of 61 patients  20 subclinical keratoconus eyes of 20 patients | N/A | Single Center (Ophthalmology Department of Torre Cardenas University Hospital, Spain). Data available upon request. | - Achieved a specificity of 93% in detecting subclinical keratoconus. | Patient Selection:  High  Index Test:  Unclear  Reference Standard:  Low  Flow and Timing:  Low |
| Shi et al., 2020(80) | Machine learning | Morphological parameters from Scheimpflug camera and ultra-high-resolution optical coherence tomography | Detected subclinical keratoconus versus normal eyes | Clinical diagnosis based on slit lamp biomicroscopy, retinoscopy, ophthalmoscopy, keratometry, topography, ocular history, and refraction. | 121 eyes of 121 patients:  50 normal eyes  38 keratoconus eyes  33 subclinical keratoconus eyes | N/A | Single Center (Affiliated Eye Hospital of Wenzhou Medical University, China). Data not available. | - Demonstrated an AUC of 0.93 in detecting subclinical keratoconus from normal eyes using both Scheimpflug camera and optical coherence tomography data. | Patient Selection:  High  Index Test:  Low  Reference Standard:  Low  Flow and Timing:  Low |
| Wan et al., 2023(81) | Fully convolutional network model with ResNet50 (FCN_ResNet50) used to conduct endothelial segmentation. Ensemble machine learning performed the classification tasks and included 4 pretrained deep learning networks: DenseNet 121, ResNet 50, Inception_v3, and MobileNet_v2 | Corneal endothelial images from specular microscopy | Detected corneal endothelium and detected keratoconus | Clinical diagnosis based on symptoms, slit lamp findings, and corneal topography. | 403 keratoconus eyes of 221 patients  370 myopic eyes of 185 patients | N/A | Single Center (Refractive Surgery Center of West China Hospital, China). Data available upon request. | - FCN_ResNet50 demonstrated an accuracy of approximately 90% for endothelial segmentation. - The ensemble machine learning model demonstrated greater than 92% accuracy and 98% AUC in detecting keratoconus using endothelial cell images. | Patient Selection:  High  Index Test:  Unclear  Reference Standard:  Low  Flow and Timing:  Unclear |
| Al-Timemy et al., 2023(82) | Xception and InceptionResNetV2 deep learning architectures used for feature extraction. Xception and InceptionResNetV2 used to identify subclinical keratoconus. Xception and InceptionResNetV2 were pretrained. | Networks were trained with sagittal, front elevation, and corneal pachymetry maps of normal/keratoconus eyes or normal/keratoconus/suspect keratoconus eyes. | Detected subclinical keratoconus, keratoconus, and normal eyes | Corneal specialists categorized eyes as keratoconus, subclinical keratoconus, or normal based on clinical diagnosis, topography, and slit lamp examination. | Dataset 1: 4113 images of 1371 eyes including 500 normal eyes, 500 suspect keratoconus eyes, 371 keratoconus eyes  Dataset 2: 639 images of 213 eyes including 114 normal eyes, 99 keratoconus eyes  Datasets were each divided into training (80%), validation (10%), and test (10%) sets. | Trained models available and linked within the manuscript. | Multicenter ((1) Al-Amal Eye Clinic, Iraq and (2) Assiut University Hospital, Egypt). Dataset available and linked within the manuscript. | - The models achieved an AUC of 0.99 in detecting normal eyes from subclinical keratoconus and keratoconus eyes using Dataset 1, and AUCs of 0.91-0.92 using Dataset 2. | Patient Selection:  High  Index Test:  Low  Reference Standard:  Low  Flow and Timing:  Low |
| Castro-Luna et al., 2020(83) | Naïve Bayes classifier | Indices from Placido-based corneal topography | Detected keratoconus versus control eyes | Clinical diagnosis based on slit lamp findings and topography. | 30 keratoconus eyes of 30 patients  30 control eyes without topographic alteration of 30 patients | N/A | Single Center (Department of Keratoconus of INVISION Ophthalmology Clinic, Spain). Data availability not specified. | - Demonstrated a sensibility and specificity of 100%. | Patient Selection:  High  Index Test:  Unclear  Reference Standard:  Low  Flow and Timing:  Low |
| Abdelmotaal et al., 2020(84) | Domain-specific convolutional neural network | Color-coded Scheimpflug camera tomography maps: front elevation, back elevation, corneal pachymetry, front sagittal curvature | Classified Scheimpflug camera color-coded tomography maps as keratoconus, subclinical keratoconus, or normal. | Two corneal specialists categorized images as keratoconus, subclinical keratoconus, or normal based on slit lamp examination, keratometry, retinoscopy, and topography. | 3218 eyes of 1669 patients  Training/validation set: 830 keratoconus images, 857 subclinical keratoconus images, 887 normal images  Test set: 208 keratoconus images, 215 subclinical keratoconus images, 221 normal eyes | N/A | Single Center (Assiut University Hospital, Egypt). Data availability not specified. | - Achieved high accuracies in classifying maps in the training set (0.983) and test set (0.953). | Patient Selection:  High  Index Test:  Unclear  Reference Standard:  Low  Flow and Timing:  Low |
| Twa et al., 2005(85) | Decision tree classifier | Zernike polynomial coefficients of the corneal surface using videokeratography data of normal and keratoconus eyes | Distinguished normal and keratoconus eyes. | Clinical diagnosis based on chart review and Collaborative Longitudinal Evaluation of Keratoconus Study criteria. | 132 normal eyes of 92 patients  112 keratoconus eyes of 71 patients | N/A | Single Center (Ohio State University, USA). Data availability not specified. | - The decision tree classifier demonstrated an accuracy and AUROC of 92% and 0.97, respectively, in distinguishing normal and keratoconus eyes using 4 Zernike polynomials: inferior elevation, greater sagittal depth, oblique toricity, and trefoil. | Patient Selection:  High  Index Test:  Low  Reference Standard:  Low  Flow and Timing:  Low |
| Elsawy et al., 2021(31) | Deep learning with parallel resolution-specific encoders for multi-resolution feature fusion, pretrained VGG16, AlexNet, and VGG19 networks | Optical coherence tomography images labelled with the diagnosis (0=Fuchs’ endothelial dystrophy, 1=healthy control, 2=keratoconus) | Output score used to build a heatmap to classify eyes as Fuchs’ endothelial dystrophy, keratoconus, or normal | Clinical diagnosis made by corneal specialists | 3677 images of 112 normal eyes of 67 patients  2297 images of 48 Fuchs’ endothelial dystrophy eyes of 30 patients  10747 images of 261 keratoconus eyes of 161 patients | N/A | Single Center (Bascom Palmer Eye Institute, USA). Data availability not specified. | - Achieved a sensitivity, specificity, and AUC of 0.94, 0.94, and 0.95, respectively, in detecting keratoconus eyes at the scan-level. | Patient Selection:  Unclear  Index Test:  Low  Reference Standard:  Low  Flow and Timing:  Low |
| Firat et al., 2022(86) | AlexNet (deep learning) for generating feature vectors. ReliefF and Laplacian algorithms used for identifying the most important features from the feature vector. Support vector machine for feature classification. | Axial/sagittal curvature, back elevation, front elevation, and corneal thickness Pentacam maps | Detected keratoconus versus healthy eyes | Clinical diagnosis based on Pentacam tomography, visual acuity, and slit lamp biomicroscopy. | 341 keratoconus eyes of 178 patients  341 healthy eyes of 178 patients | N/A | Single Center (Malatya İnönü University Ophthalmology Department, Turkey). Data available upon require. | - Achieved high accuracy (98.53%), sensitivity (98.06%), and specificity (99.01%). | Patient Selection:  Unclear  Index Test:  Unclear  Reference Standard:  Unclear  Flow and Timing: Unclear |
| Mosa et al., 2019(87) | Support vector machine and decision tree classifiers | Extracted features from sagittal, pachymetry, front and back elevation Pentacam maps | Detected keratoconus versus normal eyes | Clinical diagnosis made by specialist. | 25 normal eyes of 25 patients  15 keratoconus eyes of 15 patients | N/A | Single Center (Al-Amal Eye Private Clinic, Iraq). Data availability not specified. | - Support vector machine and decision classifier demonstrated accuracies of 90% and 87.5%, respectively. | Patient Selection:  High  Index Test:  Low  Reference Standard:  Low  Flow and Timing:  Low |
| Ambrósio et al., 2023(88) | Random forest | 10 Pentacam HR features and 8 Corvis ST parameters | Classified eyes according to the following categories: (1) normal versus disease, (2) normal versus clinical ectasia, and (3) normal versus very asymmetric ectasia with normal topography | Categorization based on slit lamp biomicroscopy, topometry, visual acuity, ophthalmic surgical history, and medication history. | 1680 normal eyes of 1680 patients  1181 keratoconus eyes of 1181 patients  551 very asymmetric ectasia with normal topography eyes of 551 patients  474 unoperated eyes with ectasia of 474 patients | N/A | Data availability not specified. | - Achieved an AUC of 0.945 and 0.999 for detecting very asymmetric ectasia with normal topography and clinical ectasia, respectively. | Patient Selection:  High  Index Test:  Low  Reference Standard:  Low  Flow and Timing:  Low |
| Lavric et al., 2020(89) | 25 machine learning models | Corneal parameters from Casia OCT instrument | Classified eyes as: (1) normal or suspect keratoconus, and (2) normal, suspect keratoconus, or keratoconus | Categorization based on the Ectasia Screening Index. | 3151 images of 3146 eyes:  1970 normal images  791 forme fruste keratoconus images  390 keratoconus images  3003 eyes for training and testing, 148 eyes for validation | N/A | Multicenter. Data publicly available. | - The quadratic support vector machine demonstrated the best performance in discriminating keratoconus, suspect keratoconus, and healthy eyes (accuracy of 93%). - The cubic support vector machine demonstrated the best performance in discriminating keratoconus and healthy eyes (accuracy of 94%). | Patient Selection:  High  Index Test:  Low  Reference Standard:  Low  Flow and Timing:  Low |
| De Almeida et al., 2021(90) | Paraconsistent feature engineering and support vector machine | 52 Pentacam tomography parameters | Generated the Corneal Tomography Multivariate Index (CTMVI) | Categorization based on visual acuity, topography, tomography, retinoscopy, keratometry, and slit lamp biomicroscopy. | 411 healthy eyes of 411 patients  302 keratoconus eyes of 302 patients  64 very asymmetric ectasia with normal corneal topography eyes of 64 patients | N/A | Single Center (Visum Eye Center, Brazil). Data availability not specified. | - CTMVI achieved a 100% sensitivity and specificity in discriminating healthy eyes from keratoconic eyes. - The index also achieved a sensitivity and specificity of 87.5% and 84.95%, respectively, in detecting very asymmetric ectasia with normal topography. | Patient Selection:  High  Index Test:  Low  Reference Standard:  Low  Flow and Timing:  Low |
| Zéboulon et al., 2020(91) | Unsupervised machine learning | Orbscan maps: elevation against the anterior best fit sphere, elevation against the posterior best fit sphere, axial anterior curvature, pachymetry | Categorized Orbscan examinations into the following clusters: normal, keratoconus, and history of refractive surgery | Two corneal topography experts labeled Orbscan examinations. | 13705 examinations of 13705 eyes of 6979 patients:  10218 normal examinations  1317 keratoconus examinations  678 history of refractive surgery examinations  235 Fuchs’ endothelial dystrophy examinations  1257 Other examinations | N/A | Single Center (Rothschild Foundation, France). Data available upon request | - Demonstrated an accuracy of 96.5% in clustering the Orbscan examinations. | Patient Selection:  High  Index Test:  Low  Reference Standard:  Low  Flow and Timing:  Low |
| Al-Timemy et al., 2021(92) | Ensemble of Deep Transfer Learning with 4 pretrained networks: SqueezeNet, AlexNet, ShuffleNet, MobileNet-v2. | Pentacam indices and the following maps: sagittal, corneal thickness, elevation front, elevation back | Detected keratoconus versus normal corneal maps based on output probabilities for each class | Clinical diagnosis made by an ophthalmologist and ophthalmology supervisor based on slit lamp biomicroscopy and corneal topography. | 219 normal cases  47 forme fruste cases  178 keratoconus cases  Data divided into training (66%), validation (12%), and test (22%) sets. | N/A | Single Center (Al-Amal Ophthalmic Center, Iraq). Data availability not specified. | - The combination of AlexNet, sagittal map, elevation back map, and the Logistic Regression with Stochastic Gradient Descent classifier for Pentacam indices demonstrated the best performance with an accuracy of 98.3%. | Patient Selection:  Unclear  Index Test:  Low  Reference Standard:  Low  Flow and Timing:  Low |
| Zaki et al., 2021(93) | Pretrained VGGNet-16 and a convolutional neural network model | Pretrained weights from the pretrained VGGNet-16 and preprocessed lateral segment photographed images | Predicted the probability of eyes belonging to the keratoconus or normal class | Clinical diagnosis made by an optometrist based on topography. | 2000 keratoconus lateral segment photographed images of 125 patients  2000 normal or non-keratoconus lateral segment photographed images of 125 patients  Data divided into training (60%), testing (20%), and validation (20%) sets. | N/A | Single Center (Hospital Kuala Lumpur, Malaysia). Data is publicly available. | - Demonstrated an accuracy, sensitivity, and specificity of 95.75%, 92.25%, and 99.25%, respectively. | Patient Selection:  Unclear  Index Test:  Low  Reference Standard:  Low  Flow and Timing:  Low |
| Alshammari et al., 2021(94) | Deep learning | OPD-Scan III corneal topography images and hyperparameters | Detected keratoconus versus normal eyes. | Not specified | 228 images of 228 healthy eyes  228 images of 228 keratoconus eyes  6609 total images (after data augmentation)  Data divided into training/validation (70%) and test (30%) sets. | N/A | Single Center (Baghdad Teaching Hospital, Iraq). Data availability not specified. | - Achieved an accuracy and sensitivity of 92.04% and 90.56%, respectively. | Patient Selection:  High  Index Test:  Low  Reference Standard:  Low  Flow and Timing: Low |
| Abdelmotaal et al., 2021(95) | Pix2pix conditional generative adversarial network (CGAN) for generating color-coded corneal tomography images. Deep convolutional neural network (DCCN) using VGG-16 network for detecting keratoconus versus normal images. | Pix2pix was trained with the original dataset of Pentacam images. DCCN was trained with the following datasets: Balanced original dataset, imbalanced original dataset, imbalanced original dataset with traditional augmentation, imbalanced original dataset partly augmented with synthesized images, imbalanced original dataset fully augmented with synthesized images, balanced synthesized dataset | Classified eyes as keratoconus, early keratoconus, or normal | Clinical diagnosis based on slit lamp biomicroscopy, keratometry, retinoscopy, topography, and tomography. | Original dataset (1778 eyes of 923 patients): 344 keratoconus images, 584 early keratoconus images, 890 normal images. 30 images from each group were used as the test set and the remaining images were used for training the pix2pix network and training/validation of the DCCN.  Synthesized dataset: 150 images each of keratoconus, early keratoconus, and normal eyes. | N/A | Single Center (Assiut University Hospital, Egypt). Data availability not specified. | - Synthetic images led to accurate discrimination between keratoconus, early keratoconus, and normal eyes by human reviewers in the study with an inter-rater agreement of 0.90 for the synthesized dataset. - The deep convolutional neural network demonstrated the highest accuracy (99.78%) when discriminating between early keratoconus and normal eyes after training with the balanced synthesized dataset. | Patient Selection:  High  Index Test:  Low  Reference Standard:  Low  Flow and Timing:  Low |
| Silverman et al., 2014(96) | Linear discriminant analysis, neural network analysis | 6 parameters from corneal epithelial and stromal thickness maps from Artemis-1 very high-frequency ultrasound arc-scans. Parameters were selected by stepwise linear discriminant analysis | Detected keratoconus versus normal eyes | Clinical diagnosis based on topography and tomography. | 130 normal eyes of 130 patients  74 keratoconus eyes of 74 patients  Data divided into training (70%) and test (30%) sets. | N/A | Single Center (London Vision Clinic, United Kingdom). Data availability not specified. | - The specificity and sensitivity of the neural network model in detecting keratoconus eyes was 99.5% and 98.9%, respectively, in the test set. | Patient Selection:  Low  Index Test:  Low  Reference Standard:  Low  Flow and Timing:  Low |
| Abdelmotaal et al., 2023(97) | Convolutional neural network based on DenseNet121 | 3-dimensional pseudoimage representations from corneal deformation videos | Detected keratoconus versus normal eyes with a probability for each class | Clinical diagnosis based on corneal topography. | Dataset 1 (external validation set): 131 keratoconus eyes and 101 normal eyes  Dataset 2 (70% training/validation, 30% test): 243 normal eyes and 259 normal eyes | N/A | Multicenter ((1) Hospital de Olhos-CRO, Brazil and (2) Salouti Eye Center, Iran). Data availability not specified. | - Achieved an AUC and accuracy of 0.93 and 0.88, respectively, in detecting keratoconus in the external validation set. | Patient Selection:  High  Index Test:  Low  Reference Standard:  Low  Flow and Timing:  Low |
| Francis et al., 2023(98) | Decision tree with sigmoid calibrated classifier | Preoperative Corvis ST parameters | Detected normal versus ectasia eyes post-small-incision lenticule extraction (SMILE) | Preoperative and postoperative Corvis ST measurements | 10 post-SMILE ectasia eyes  32 post-SMILE normal eyes | N/A | Multicenter ((1) Narayana Nethralaya Eye Hospital and Sankara Nethralaya, India and (2) Humanitas Clinical and Research Center, Italy). Data availability not specified. | - Demonstrated an AUROC of 1.0. | Patient Selection:  High  Index Test:  Low  Reference Standard:  Low  Flow and Timing:  Low |
| Velázquez-Blázquez et al., 2020(99) | Ordinal logistic regression model | 27 demographic, clinical, pachymetric, and geometric parameters | Classified eyes as healthy, mild keratoconus, or early keratoconus | Grading based on the RETICS classification system | Training set (178 eyes of 178 patients): 74 healthy eyes, 104 keratoconus eyes (61 early keratoconus, 43 mild keratoconus)  Validation set (41 eyes of 41 patients): 19 healthy eyes, 22 keratoconus eyes (14 early keratoconus, 8 mild keratoconus) | N/A | Single Center (Vissum Corporation Alicante, Spain). Data availability not specified. | - Demonstrated an accuracy of 79%, 80%, and 69% in detecting normal, mild keratoconus, and early keratoconus eyes, respectively, in the validation set. | Patient Selection:  High  Index Test:  Low  Reference Standard:  Low  Flow and Timing:  Low |
| Santhiago et al., 2022(100) | Machine learning with LightGBM algorithm and diagram t-distributed stochastic neighbor embedding | 20 risk factors (14 of which were derived from feature engineering) | Predicted the probability of the patient developing postoperative ectasia | Clinical diagnosis based on postoperative change in inferior steepening, myopia, astigmatism, and visual acuity. | 65 eyes developing ectasia after laser in situ keratomileusis (LASIK) (ectasia)  274 eyes not developing ectasia after LASIK  All eyes had normal preoperative topography | N/A | Multicenter ((1) University of São Paulo, Brazil, (2) Federal University of Minas Gerais, Brazil, and (3) Hadassah Medical Center, Israel). Data availability not specified. | - The best performing model used corneal thickness, percent tissue altered (PTA), derived PTA, and age weighted value to perform the classification task and demonstrated a recall, precision, and AUROC of 0.98, 0.77, and 0.99, respectively. | Patient Selection:  High  Index Test:  Unclear  Reference Standard:  Unclear  Flow and Timing:  Low |
