# Supplementary material for: Utility of artificial intelligence in the diagnosis and management of keratoconus: a systematic review
Source: Front Ophthalmol (Lausanne). 2024 May 17;4:1380701. doi: 10.3389/fopht.2024.1380701 (PMC11182163; doi:10.3389/fopht.2024.1380701)
Supplement: Supplementary file 2 [file Table_2.docx]

**Supplemental Table 2.** Original research studies for the application of artificial intelligence in the etiology and pathogenesis of keratoconus and other corneal ectasias.

| **Author, Year** | **Type of AI** | **Input used for training** | **Output** | **Ground Truth/Reference Standard** | **Dataset size** | **Availability of Algorithm/Model** | **Availability of Dataset** | **Major Study Results** |
| --- | --- | --- | --- | --- | --- | --- | --- | --- |
| Hosoda et al., 2020(109) | IBM’s Watson for Drug Discovery | 8 previously identified keratoconus susceptibility genes and STON2 rs2371597 | Determined connections between genes associated with keratoconus as well as ranked genes by similarity. | Not reported | 179 keratoconus participants  9589 control participants | IBM no longer sells this AI model. | Control samples are from the publicly available Tohoku Medical Megabank Project. | - STON2 rs2371597 was significantly associated with keratoconus development. - SMAD3 rs12913547 was identified as a susceptibility locus. |
| Wang et al., 2022(110) | Random forest model, support vector machine model, generalized linear model | Transcriptome expression profiles from human corneas | Determined differentially expressed genes | Not reported | 26 participants from the GSE151631 dataset  50 participants from the GSE77938 dataset  20 participants from the GSE112155 dataset (verification dataset) | N/A | Datasets available from the National Center for Biotechnology Information Gene Expression Database. | - 8 differentially expressed genes (AREG, BBC3, DUSP2, MAP3K8, SMAD7, CDKN1A, JUN, and LIF) were identified between patients with and without keratoconus. |
| Nokas et al., 2023(111) | Support vector machine, decision tree, random forest, XGBoost | Feature vector generated from accelerometer and gyroscope data detected by a wrist-mounted sensor worn by users | Reminded user to stop eye rubbing if the model detected eye rubbing hand movement | Actual random activity versus eye rubbing movements | Two participants: one healthy participant, one participant with keratoconus  60 minutes of free activities with 60 eye rubbing movements in training set  40 minutes of free activities with 40 eye rubbing movements in test set | N/A | Data available upon request. | - Demonstrated an accuracy of 0.99 in reminding the user to stop rubbing their eyes when the user was rubbing their eyes. |
