# Supplementary material for: Utility of artificial intelligence in the diagnosis and management of keratoconus: a systematic review
Source: Front Ophthalmol (Lausanne). 2024 May 17;4:1380701. doi: 10.3389/fopht.2024.1380701 (PMC11182163; doi:10.3389/fopht.2024.1380701)
Supplement: Supplementary file 3 [file Table_3.docx]

**Supplemental Table 3.** Original research studies for the application of artificial intelligence in severity and clinical grading of keratoconus and other corneal ectasias.

| **Author, Year** | **Type of AI** | **Input used for training** | **Output** | **Ground Truth/Reference Standard** | **Dataset size** | **Availability of Algorithm/Model** | **Availability of Dataset** | **Major Study Results** | **Risk of Bias Assessment** |
| --- | --- | --- | --- | --- | --- | --- | --- | --- | --- |
| Herber et al., 2021(113) | Supervised machine learning algorithms: Linear discriminant analysis and random forest algorithms | Dynamic corneal response and corneal thickness related (pachymetric) parameters from Corvis ST | Predicted keratoconus stage (healthy, mild, moderate, advanced) | Staging based on topographical keratoconus classification. | 116 healthy eyes of 116 patients  318 keratoconus eyes of 318 patients  Divided data into training (70%) and validation (30%). | N/A | Single Center (University Hospital Carl Gustav Carus, Germany). Data available e upon request. | - The accuracy of linear discriminant analysis in predicting the severity of keratoconus (71%) was lower than that of the random forest algorithm (78%). | Patient Selection:  Low  Index Test:  Low  Reference Standard:  Low  Flow and Timing:  Low |
| Yousefi et al., 2018(114) | Unsupervised machine learning involving principal component analysis, manifold learning, and density-based clustering | Corneal topography, elevation, and pachymetry parameters | Identified keratoconus stage | Ectasia Screening Index (ESI) | 1970 healthy eyes  796 forme fruste keratoconus eyes  390 keratoconus eyes | Code available on GitHub and linked within the manuscript. | Multicenter. Dataset publicly available on Harvard Dataverse. | - The model identified four clusters: (1) normal eyes, (2) mostly healthy eyes and eyes with forme fruste keratoconus, (3) mild keratoconus, and (4) advanced keratoconus. | Patient Selection:  High  Index Test:  Low  Reference Standard:  Low  Flow and Timing:  Unclear |
| Mahmoud et al., 2021(115) | Machine learning | 2-dimensional frontal and lateral ocular images | 3-dimensional images were the output and used to detect the corneal curvature and stage keratoconus severity | Clinical diagnosis made by experts | Training set: 250 keratoconus and 200 normal images  Validation set: 58 mild keratoconus, 70 moderate keratoconus, 40 severe keratoconus, 100 normal corneal images | N/A | Dataset available on GitHub. | - Demonstrated an accuracy of 97.8%. | Patient Selection:  High  Index Test:  Unclear  Reference Standard:  Low  Flow and Timing:  Low |
| Issarti et al., 2020(116) | Feedforward neural network | Corneal elevation and minimum thickness from Pentacam HR | Produced a logistic index for scoring keratoconus severity | Clinical diagnosis based on slit lamp findings and corneal tomography findings | 812 eyes of 812 patients:  304 eyes with normal topography  117 suspect keratoconus eyes  34 early keratoconus eyes  158 mild keratoconus eyes  199 moderate to advanced keratoconus eyes  Divided data into training (70%) and validation (30%). | N/A | Multicenter ((1) Antwerp University Hospital, Belgium and (2) Ghent University Hospital, Belgium). Data availability not specified. | - The machine learning model demonstrated superior accuracy (99.9%) for grading keratoconus relative to Belin/Ambrósio Display Deviation (98.2%) and the Pentacam Topographical Keratoconus Classification (94.7%). | Patient Selection:  High  Index Test:  Low  Reference Standard:  Low  Flow and Timing:  Low |
| Aatila et al., 2021(117) | Machine learning with 11 feature selection algorithms | 446 corneal parameters | Detected keratoconus versus normal eyes and staged keratoconus severity | Keratoconus dataset published in Harvard Dataverse | 264 normal eyes  2595 healthy eyes with forme fruste keratoconus  221 mild keratoconus eyes  82 advanced keratoconus eyes | N/A | Publicly available in Harvard Dataverse | - The sequential forward selection algorithm selected 10 variables out of the 446 parameters for the classification tasks. - Using these 10 variables, the random forest AI model achieved the best accuracy in detecting keratoconus (98%) and staging keratoconus severity (95%). | Patient Selection:  Unclear  Index Test:  Low  Reference Standard:  Low  Flow and Timing:  Unclear |
| Ghaderi et al., 2021(104) | Ensemble learning model | 19 parameters from Pentacam regarding corneal curvature, eccentricity, anterior chamber, corneal volume, and pachymetry | Classified eyes as normal or keratoconus. Staged keratoconus eyes according to severity. | Classified eyes based on topography | 450 eyes of 450 patients:  130 normal eyes  120 early keratoconus  105 Moderate keratoconus  95 Advanced keratoconus  Data divided into training (80%) and validation (20%) for test | Code not publicly available | Single Center (Noor Medical Eye Clinic, Iran). Data availability not specified. | - The model combined with multilayer perceptron and neuro-fuzzy system classifiers, as well as the Naïve Bayes combination method, demonstrated an accuracy, sensitivity, and specificity of 98.2%, 99.1%, and 96.2%, respectively, in detecting keratoconus. - In classifying eyes across all four groups, the accuracy, sensitivity, and specificity was 98.2%, 98.5%, and 99.4%, respectively. | Patient Selection:  High  Index Test:  Low  Reference Standard:  Low  Flow and Timing:  Low |
| Malyugin et al., 2021(118) | Machine learning techniques including principal component analysis and clustering | Seven parameters selected by principal component analysis: index of surface variance, index of vertical asymmetry, keratoconus index, index of height decentration, maximum curvature power on corneal front surface, inferior-superior value, and minimal sagittal curvature | Predicted the presence and stage of keratoconus | Clinical diagnosis and staging done by ophthalmologists based on keratotopography data and medical records. | 400 normal eyes  400 eyes with one of the following: preclinical keratoconus, stage 1, stage 2, or stage 3 keratoconus  52 stage 4 keratoconus  Data was divided into training (60%) and testing (40%). | N/A | Multicenter (S. Fyodorov Eye Microsurgery Complex Head Office, Russia and 2 associated branches). Data available with permission. | - Demonstrated an AUC of 0.98, 0.95, 0.96, 0.97, 0.97, and 1.00 for detecting normal, preclinical keratoconus, stage 1, stage 2, stage 3, and stage 4 keratoconus eyes, respectively. | Patient Selection:  Unclear  Index Test:  Low  Reference Standard:  Low  Flow and Timing:  Low |
| Tai et al., 2022(119) | Machine learning using shape-based clustering and feature-based clustering | Legendre modes from the corneal deformation response images from Corvis ST | Determined associations with keratoconus severity and weak regions of the cornea | Keratoconus severity determined from the Keratoconus Index and Keratoconus Severity Index | 31 keratoconus eyes of 31 patients | N/A | Single Center (National Taiwan University Hospital, Taiwan). Data availability not specified. | - Demonstrated that symmetrical modes of the corneal deformation response were strongly correlated to keratoconus severity while asymmetric modes of the response were strongly correlated to the weak centroid. | Not applicable |
| Kamiya et al., 2021(120) | Deep learning | VGG-16 model pretrained with color-coded maps of anterior and posterior elevation, anterior and posterior curvature, total refractive power, and pachymetry maps. | Detected keratoconus and graded keratoconus severity. | Clinical diagnosis and staging completed by corneal specialists using slit lamp examination, topography, and the Amsler-Krumeich classification. | 170 healthy eyes  54 Grade 1 keratoconus eyes  52 Grade 2 keratoconus eyes  23 Grade 3 keratoconus eyes  50 Grade 4 keratoconus eyes | N/A | Single Center (Miyata Eye Hospital, Japan). Data available upon request. | - Achieved an accuracy, sensitivity, and specificity of 0.966, 0.988, and 0.944, respectively, in detecting keratoconus. - With staging keratoconus, the model demonstrated an accuracy of 0.785. | Patient Selection:  High  Index Test:  Low  Reference Standard:  Low  Flow and Timing:  Low |
| Chen et al., 2021(121) | Convolutional neural networks | 4 color-coded corneal tomography maps: axial, anterior and posterior elevation, pachymetry | Detected keratoconus versus normal eyes and determined keratoconus stage. | Classification of images using Amsler-Krumeich grading. | 134 scans of healthy eyes  282 scans of Stage 1 keratoconus eyes  425 scans of Stage 2 keratoconus eyes  208 scans of Stage 3 keratoconus eyes  877 scans of Stage 4 keratoconus eyes  Data divided into training (80%) and testing (20%) sets. 20% of the training set was used as the validation set. | Code not available | Multicenter ((1) The Royal Liverpool University Hospital, Iran, (2) Sedaghat Eye Clinic, Iran and (3) the New Zealand National Eye Center, New Zealand). data not available. | - Using all 4 maps, the model demonstrated an accuracy of 0.9785 among testing set images. - The model was also able to distinguishealthy eyes and stage 1 eyes, as well as stage 1 and stage 2 eyes, with an accuracy of 0.90 and 0.9032, respectively. | Patient Selection:  High  Index Test:  Low  Reference Standard:  Low  Flow and Timing:  Low |
| Dos Santos et al., 2019(122) | CorneaNet (neural network) | Ultra-high-resolution optical coherence tomography images of healthy and keratoconus eyes | Generated thickness maps of corneal epithelium, Bowman’s layer, and stroma | True labels for each pixel | 20,160 images of 72 healthy eyes of 36 patients and 70 keratoconus eyes of 57 patients | N/A | Single Center (University of Vienna, Austria). Data availability not specified. | - CorneaNet demonstrated a validation accuracy of 99.6% and segmentation time less than 25 milliseconds for segmentation of the cornea into epithelium, Bowman’s layer, and stroma on optical coherence tomography images. | Not applicable |
| Dong et al., 2022(123) | Deep learning | Anterior segment-optical coherence tomography | Determined thickness of epithelial and corneal tissues | Three ophthalmologists labeled boundaries of the cornea and epithelial layer | 1430 images of 715 eyes:  118 normal eyes  134 mild keratoconus eyes  239 moderate keratoconus eyes  152 severe keratoconus eyes  71 scarring keratoconus eyes | Code available on GitHub. | Single Center (Qingdao Eye Hospital of Shandong First Medical University, China). Data available upon request. | - Measured tissue thickness with an error less than 4 microns. - The corneal thickness decreased with increasing keratoconus severity. | Not applicable |
| Lavric et al., 2021(124) | 23 machine learning algorithms | 18 corneal elevation, 10 topography, and 10 pachymetry parameters from Pentacam | Staged keratoconus severity | Previous clinical diagnosis | 5881 eyes of 2800 patients:  1726 healthy eyes  345 stage 1 keratoconus eyes  1380 stage 2 keratoconus eyes  1800 stage 3 keratoconus eyes  630 keratoconus eyes | N/A | Single Center (Federal University of São Paulo, Brazil). Data available upon request. | - The support vector machine model was capable of distinguishing keratoconus severity levels with an area under the receiver operating characteristic curve of 0.88 using minimum curvature radius, as well as eccentricity and asphericity of the cornea. | Patient Selection:  High  Index Test:  Low  Reference Standard:  Low  Flow and Timing:  Low |
| Bolarín et al., 2020(125) | 2 machine learning models: (1) multivariate logistic regression for detecting keratoconus, (2) ordinal logistic regression for staging keratoconus | Age, gender, 7 optical parameters, 5 pachymetry parameters, 3 morpho-geometric parameters | Detected keratoconus and staged keratoconus severity | Clinical diagnosis based on topography, visual acuity, manifest refraction, slit lamp biomicroscopy, Goldmann tonometry, fundus exam, and pachymetry. | 62 healthy eyes of 62 patients  107 keratoconus eyes of 107 patients including 44 grade 1 eyes, 18 grade 2 eyes, 15 grade 3 eyes, 15 grade 4 eyes, and 15 grade 5 eyes  Data divided into training (63.2%) and validation (36.8%). | N/A | Single Center (Vissum Corporation Alicante, Spain). Data availability not specified. | - The ordinal logistic regression model demonstrated an accuracy of 0.698 in staging keratoconus severity. | Patient Selection:  High  Index Test:  Low  Reference Standard:  Low  Flow and Timing:  Low |
| Zorto et al., 2023(126) | 8 machine learning techniques | Patient risk factors, Pentacam data, individually and combined | Classified keratoconus severity | Severity classification using the Amsler-Krumeich criteria. | 140 Grade 1 cases  51 Grade 2 cases  24 Grade 3 cases  22 Grade 4 cases  Data divided into training (90%) and test (10%) sets. | N/A | Single Center (Vision Eye Institute Chatswood, Australia). Data availability not specified. | - The machine learning model using a decision tree algorithm demonstrated an accuracy of 75%, 79%, and 100% using risk factor data, Pentacam data, and both types of data combined, respectively, in classifying keratoconus severity. | Patient Selection:  Unclear  Index Test:  Low  Reference Standard:  Low  Flow and Timing:  Low |
| Smolek et al., 1997(127) | 2 neural networks: (1) classification network for the detection of keratoconus and subclinical keratoconus, (2) cone severity network for staging keratoconus severity based on conelike qualities | 10 indexes from TMS-1 topography | Detected and graded the severity of keratoconus. Classification network outputs included KC (keratoconus), KCS (suspect keratoconus, and OTHER. Cone severity network outputs included 0 (other), 0.25 (suspect keratoconus), 0.5 (mild keratoconus), 0.75 (moderate keratoconus), and 1 (advanced keratoconus). | Clinical diagnosis based on medical record review and review of topography of keratoconus suspects by the authors of the study. Staging of keratoconus based on map contour power and disrupted contour steps. | 300 TMS-1 topography maps (Divided into 50% training and 50% testing)  Test set: 6 keratoconus suspect, 11 mild keratoconus, 13 moderate keratoconus, 9 advanced keratoconus, 111 other | N/A | Single center (Louisiana State University Eye Center, USA). Data availability not specified. | - The classification neural network demonstrated perfect accuracy, specificity, and sensitivity for detecting keratoconus. - There was a strong correlation between the severity network results and keratoconus severity based on keratoconus prediction index (R=0.892, p<0.0001). | Patient Selection:  High  Index Test:  Low  Reference Standard:  Low  Flow and Timing:  Unclear |
