# Supplementary material for: Utility of artificial intelligence in the diagnosis and management of keratoconus: a systematic review
Source: Front Ophthalmol (Lausanne). 2024 May 17;4:1380701. doi: 10.3389/fopht.2024.1380701 (PMC11182163; doi:10.3389/fopht.2024.1380701)
Supplement: Supplementary file 4 [file Table_4.docx]

**Supplemental Table 4.** Original research studies for the application of artificial intelligence in predicting progression of keratoconus and other corneal ectasias.

| **Author, Year** | **Type of AI** | **Input used for training** | **Output** | **Ground Truth/Reference Standard** | **Dataset size** | **Availability of Algorithm/Model** | **Availability of Dataset** | **Major Study Results** | **Risk of Bias Assessment** |
| --- | --- | --- | --- | --- | --- | --- | --- | --- | --- |
| Kundu et al., 2023(129) | Two models based on either tomographic changes or clinical risk factors using random forest classifier | Differences in Pentacam corneal tomography parameters between two visits, as well as clinical and ocular surface risk factors from patient questionnaire | Predicted progression versus no progression among keratoconus eyes | Measured change in maximum anterior surface curvature between two visits at least 6 months apart | 900 images of 450 keratoconus eyes (450 patients) | N/A | Single Center (Eye hospital in Bengaluru, India). Data availability not specified. | - The AI model based on clinical risk factors demonstrated an area under the curve (AUC) of 0.812 - 76% of the cases classified as progression by the tomographic changes AI model were also categorized as progression by the clinical risk factors AI model. | Participants:  Unclear  Index Test:  Low  Outcome:  Low  Flow and Timing:  Low  Analysis:  Low |
| Shetty et al., 2021(130) | Three AI models trained with different values of increase in maximum anterior curvature (Model A: 0.75 D, Model B: 1.00 D, Model C: 1.25 D) | Changes in Pentacam parameters including increase in maximum anterior curvature | Categorized eyes as “progression” or “no progression” | The authors determined the classification of each eye based on a predetermined maximum curvature of the anterior surface. | 1884 images of 366 eyes (296 patients) | N/A | Single Center (Narayana Nethralaya Eye Hospital, India). Data availability not specified. | - Model C demonstrated the best performance with an AUC, sensitivity, specificity, and classification accuracy of 0.93, 89%, 81%, and 91%, respectively. | Participants:  Low  Index Test:  Low  Outcome:  Low  Flow and Timing:  Low  Analysis:  Low |
| Reddy et al., 2022(131) | Deep learning neural network | Maps from Scheimpflug analyzer: axial/sagittal curvature, posterior elevation, anterior elevation, corneal thickness | Predicted latent progression | Keratometric progression defined by a change in flat or steep K of at least 1 D | Training and testing set: 385 eyes of 351 patients  Prediction set: 1331 eyes of 828 patients | N/A | Single Center (LV Prasad Eye Institute, India). Data availability not specified. | - The AI model detected latent progression 11.1 months earlier than the use of keratometric progression. | Participants:  High  Index Test:  Low  Outcome:  Low  Flow and Timing:  Low  Analysis:  Low |
| Jiménez-García et al., 2021(132) | Time delay neural network | Age, average keratometry, steepest radius, best fit sphere, average radius of back surface, and the logistic index for keratoconus severity | Classified eye as stable or suspect progressive | Clinical diagnosis based on corneal tomography and an ophthalmological examination. | 1155 eyes of 743 keratoconus patients | Code for models may be available upon request. | Multicenter (Retrospective Digital Computer Analysis of Keratoconus Evolution organized through the European Vision Institute Clinical Research Network). Data may be available upon request. | - The AI model demonstrated a sensitivity, specificity, positive predictive value, and negative predictive value of 70.8%, 80.6%, 71.4%, and 80.2%, respectively. | Participants:  Low  Index Test:  Low  Outcome:  Low  Flow and Timing:  Unclear  Analysis:  Low |
| Kamiya et al., 2021(133) | Deep learning | 6 color-coded maps from swept-source anterior segment optical coherence tomography: anterior elevation, anterior curvature, posterior elevation, posterior curvature, total refractive power, and pachymetry. | Predicted progressive versus non-progressive keratoconus | Clinical diagnosis made by corneal specialists based on color-coded maps and slit lamp examination. Progression was based on increase in maximum anterior curvature or worsening of visual acuity with an increase in astigmatism. | 156 image sets of 156 progressive keratoconus eyes of 156 patients  62 image sets of 62 non-progressive keratoconus eyes of 62 patients | N/A | Multicenter ((1) Miyata Eye Hospital, Japan and (2) Tokyo University Hospital, Japan). Data no longer available upon request. | - The posterior elevation map achieved the best accuracy of 0.798. | Participants:  Low  Index Test:  Low  Outcome:  Low  Flow and Timing:  Low  Analysis:  Low |
| Kato et al., 2021(134) | Convolutional neural network | The neural network was trained with age and the following maps individually and combined: axial map of the frontal corneal plane, and pachymetry map. | Predicted progression versus non-progression among keratoconus eyes. | Clinical diagnosis made by corneal specialists based on corneal tomography or topography using CASIA and Pentacam HR devices, respectively. Eyes were classified as progression versus nonprogression based on whether they underwent corneal crosslinking. | 274 keratoconus eyes of 158 patients:  90 keratoconus eyes with progression  184 keratoconus eyes without progression | Codes for training and analysis are available as supplementary material. | Single Center (Keio University School of Medicine, Japan). Data available as supplementary material. | - The combination of age, axial map of the frontal plane, and pachymetry map resulted in an AUC, sensitivity, and specificity of 0.814, 77.8%, and 69.6%, respectively. | Participants:  Unclear  Index Test:  Low  Outcome:  Low  Flow and Timing:  Low  Analysis:  Low |
| Cao et al., 2023(135) | Unsupervised machine learning | Change in Pentacam parameters from baseline by the 6-month follow-up visit | Generated clusters of keratoconus progression patterns | Medical record review used to establish diagnosis, which was based on corneal tomography changes | Training set: 556 keratoconus eyes of 362 patients, 60 control eyes of 44 patients  Validation of model at 6-months set: 314 keratoconus eyes of 228 patients, 18 control eyes of 13 patients  Validation of model at 12- and 18-months set: 381 keratoconus eyes of 253 patients, 41 control eyes of 34 patients | N/A | Single Center (Royal Victorian Eye and Ear Hospital, Australia). Data availability not specified. | - Identified three clusters of keratoconus progression patterns: fast, slow, and limited change at 6, 12, and 18 months of follow up. - There was a change in 39 curvature parameters between the three clusters. | Participants:  High  Index Test:  Low  Outcome:  Low  Flow and Timing:  Low  Analysis:  Low |
