# Supplementary material for: Utility of artificial intelligence in the diagnosis and management of keratoconus: a systematic review
Source: Front Ophthalmol (Lausanne). 2024 May 17;4:1380701. doi: 10.3389/fopht.2024.1380701 (PMC11182163; doi:10.3389/fopht.2024.1380701)
Supplement: Supplementary file 5 [file Table_5.docx]

**Supplemental Table 5.** Original research studies for the application of artificial intelligence in predicting the response to treatment/management of keratoconus and other corneal ectasias.

| **Author, Year** | **Type of AI** | **Input used for training** | **Output** | **Ground Truth/Reference Standard** | **Dataset size** | **Availability of Algorithm/Model** | **Availability of Dataset** | **Major Study Results** | **Risk of Bias Assessment** |
| --- | --- | --- | --- | --- | --- | --- | --- | --- | --- |
| Fariselli et al., 2020(136) | Artificial neural network (ANN) | 75 successful cases as defined by an improvement in visual acuity, decrease in spherical equivalent, or a decrease in corneal aberrations. | Predicted the outcome after intracorneal ring segment implantation. | Measured postoperative spherical equivalent, and corneal aberrations | 40 keratoconus eyes (50% in the artificial neural network group (18 patients), 50% in the manufacturer’s nomogram group (17 patients)) | N/A | Single Center (Vissum Institution Alicante, Spain). Data available upon request. | - ANN used to predict the results of intrastromal corneal ring segment implantation in keratoconus eyes resulted in improved visual acuity and optical quality as well as reduced spherical equivalent compared to implantation guided by the manufacturer’s nomograms. | Participants:  High  Index Test:  Low  Outcome:  Low  Flow and Timing:  Low  Analysis:  Low |
| Yousefi et al., 2020(137) | Unsupervised machine learning | Corneal parameters from optical coherence tomography (OCT) images were used to produce 2-dimensional t-distributed stochastic neighbor embedding maps | Predicted the need for future keratoplasty intervention | Proportion of the number of the eyes that underwent postoperative keratoplasty per AI-identified cluster | 3318 corneal OCT images + 333 eyes that underwent post-operative keratoplasty | N/A | Multicenter study in Japan. Data not available or will be made available upon request. | - Identified five clusters of likelihoods for the need for future keratoplasty: 2.2%, 1.0%, 33.1%, 32.7%, and 31.0%. | Participants:  Low  Index Test:  Low  Outcome:  Low  Flow and Timing:  Low  Analysis:  Low |
| Valdés-Mas et al., 2014(138) | Artificial neural network using multilayer perceptron | Topographical variables (K1, K2), surgery characteristics (incision, depth), ring characteristics (number, thickness, arc length, ring type) | Predicted postoperative corneal curvature and astigmatism | Postoperative measurements | 194 eyes of 154 patients. Data divided into training (2/3) and validation (1/3). | N/A | Single Center (Fundación Oftalmológica del Mediterráneo, Spain). Data availability not specified. | - Demonstrated an error of 0.97 D and 0.93 D for corneal curvature and astigmatism, respectively. | Participants:  Unclear  Index Test:  Low  Outcome:  Low  Flow and Timing:  Low  Analysis:  Low |
| Liu et al., 2023(139) | Machine learning models: XGBoost, CatBoost, LightGBM | 26 preoperative demographic and ophthalmic variables, 5 incorporation variables, actual changes in visual acuity and keratometry | Predicted changes in visual acuity and keratometry two years after corneal crosslinking | Two ophthalmologists validated the Pentacam images and data. Ground truth was actual postoperative changes in visual acuity and keratometry. | 277 eyes of 195 patients in training and testing sets  43 eyes of 35 patients in validation set | N/A | Single Center (Aier Eye Hospital of Wuhan University, China). Data available upon request. | - XGBoost demonstrated the best performance among the three machine learning models. | Participants:  Low  Index Test:  Low  Outcome:  Low  Flow and Timing:  Low  Analysis:  Unclear |
| Lyra et al., 2018(140) | Machine learning with linear regression | Age, Ferrara intracorneal ring segment characteristics, 37 Pentacam parameters | Predicted postoperative asphericity and average keratometry | Actual preoperative and postoperative asphericity and average keratometry | 209 keratoconus eyes of 160 patients with intrastromal corneal ring segment implants | N/A | Multicenter study from the Brazilian Study Group of Artificial Intelligence on Ocular Solutions. Data availability not specified. | - Demonstrated a lower mean absolute error by 0.11 for asphericity and 0.09 for mean keratometry relative to the nomograms. | Participants:  Unclear  Index Test:  Low  Outcome:  Low  Flow and Timing:  Low  Analysis:  Low |
| Firat et al., 2022(141) | U-Net-based 2D regression architecture | Pre- and post-treatment Pentacam images | Generated Pentacam image of disease stage in the future | Preoperative and postoperative data after 6 months | 125 keratoconus eyes of 96 patients who underwent corneal crosslinking: 1000 images for training, 125 images for testing, 125 images for validation | N/A | Single Center (Inonu University Turgut Ozal Medical Center, Turkey). Data availability not specified. | - Predicted postoperative images with a structural similarity index measure, peak signal-to-noise ratio, and root mean square error of 0.8266, 65.85, and 0.134, respectively. | Not applicable |
